# CRL4^Mahj^ E3 ubiquitin ligase promotes neural stem cell reactivation

**DOI:** 10.1371/journal.pbio.3000276

**Published:** 2019-06-06

**Authors:** Phuong Thao Ly, Ye Sing Tan, Chwee Tat Koe, Yingjie Zhang, Gengqiang Xie, Sharyn Endow, Wu-Min Deng, Fengwei Yu, Hongyan Wang

**Affiliations:** 1 Neuroscience and Behavioural Disorders Programme, Duke-NUS Medical School, Singapore, Singapore; 2 Genome Institute of Singapore, Singapore, Singapore; 3 Department of Biological Science, Florida State University, Tallahassee, Florida, United States of America; 4 Department of Cell Biology, Duke University Medical Centre, Durham, North Carolina, United States of America; 5 Temasek Life Sciences Laboratory, Singapore, Singapore; 6 Department of Biological Sciences, National University of Singapore, Singapore, Singapore; 7 NUS Graduate School for Integrative Sciences and Engineering, National University of Singapore, Singapore, Singapore; 8 Department of Physiology, Yong Loo Lin School of Medicine, National University of Singapore, Singapore, Singapore; The Francis Crick Institute, UNITED KINGDOM

## Abstract

The ability of neural stem cells (NSCs) to transit between quiescence and proliferation is crucial for brain development and homeostasis. *Drosophila* Hippo pathway maintains NSC quiescence, but its regulation during brain development remains unknown. Here, we show that CRL4^Mahj^, an evolutionarily conserved E3 ubiquitin ligase, is essential for NSC reactivation (exit from quiescence). We demonstrate that damaged DNA-binding protein 1 (DDB1) and Cullin4, two core components of Cullin4-RING ligase (CRL4), are intrinsically required for NSC reactivation. We have identified a substrate receptor of CRL4, Mahjong (Mahj), which is necessary and sufficient for NSC reactivation. Moreover, we show that CRL4^Mahj^ forms a protein complex with Warts (Wts/large tumor suppressor [Lats]), a kinase of the Hippo signaling pathway, and Mahj promotes the ubiquitination of Wts. Our genetic analyses further support the conclusion that CRL4^Mahj^ triggers NSC reactivation by inhibition of Wts. Given that Cullin4B mutations cause mental retardation and cerebral malformation, similar regulatory mechanisms may be applied to the human brain.

## Abstract 2

During the transition from quiescence to reactivation of neural stem cells, the E3 ubiquitin ligase CRL4Mahj promotes their reactivation by inhibiting Wts, a core kinase of Hippo signalling pathway.

## Introduction

The ability of stem cells to switch between quiescence and proliferation is crucial for tissue homeostasis and regeneration. Most adult neural stem cells (NSCs) exist in quiescence, a reversible dormant state, without proliferation or differentiation [1]. In response to physiological stimuli, quiescent NSCs can exit from quiescence and become reactivated to generate new neurons [2]. Whereas excessive reactivation will exhaust the NSC population prematurely, failure in NSC reactivation is associated with neurogenesis deficits and neurodevelopmental and neurodegenerative disorders [3]. However, how NSCs transit between quiescence and reactivation remains poorly understood.

Recently, *Drosophila* NSCs, called neuroblasts, have emerged as a powerful model to study mechanisms underlying NSC reactivation *in vivo*. Most *Drosophila* NSCs enter quiescence at the end of embryogenesis and resume proliferation shortly after larval hatching (ALH) [4–6]. NSC quiescence entry is regulated by intrinsic factors including Hox genes; temporal identity factors; a homeodomain transcription factor, Prospero; and a pseudokinase, Tribbles (Trbl) [7–9]. In contrast, NSC reactivation requires both extrinsic cues and intrinsic regulators. Dietary amino acids are sensed by fat body, the invertebrate equivalent of liver and adipose tissues, which stimulates blood-brain-barrier glia to secrete *Drosophila* insulin-like peptides (DILPs), activating the conserved Insulin receptor (InR)/phosphoinositide 3-kinase (PI3K)/AKT pathway to induce NSC reactivation [6,10,11]. While blood-brain-barrier glia synchronize NSC reactivation via calcium oscillations and gap junctions, cortex glia undergo remodeling to promote neuronal survival [12,13]. Moreover, spindle matrix complex such as Chromator intrinsically promotes NSC reactivation downstream of the InR/PI3K/AKT pathway [14]. In addition, glia secrete glycoprotein Anachronism to maintain NSC quiescence by an unknown mechanism [15].

Nutritional cues and glia also promote NSC reactivation by suppressing Hippo pathway, a highly conserved pathway for organ size restraint and tumor suppression [16]. The intercellular interaction of the transmembrane proteins Crumbs and Echinoid that are expressed in both NSCs and glia activates the Hippo pathway core kinases, including Hippo and Warts (Wts). Active Wts phosphorylates and inactivates transcriptional coactivator Yorkie (Yki) to suppress transcription of target genes for cellular growth and proliferation, thereby maintaining NSC quiescence [17,18]. Although the function of the Hippo pathway has been elucidated, the mechanisms regulating the turnover of its core components during NSC reactivation remain unknown.

Cullin4-RING ligase (CRL4) is an evolutionarily conserved ubiquitin ligase (E3) family that regulates protein turnover by ubiquitination followed by proteasome-mediated degradation [19]. The core components of CRL4 consist of the scaffold Cullin4 (Cul4), the adaptor damaged DNA-binding protein 1 (DDB1), and the catalytic subunit RING of Cullin (ROC) that binds to E2 enzymes [20,21]. DDB1 binds to a subset of WD40 proteins referred to as DDB1-Cul4 associated factors (DCAFs) for substrate recognition [22,23]. In *Drosophila*, CRL4 E3 is implicated in regulating multiple cellular processes, such as DNA replication, cell cycle progression, proliferation, and circadian rhythm [24–28]. Mutations of human Cullin4B (Cul4B) are associated with mental retardation and cortical malformations [29–32], suggesting that Cul4B is important for brain development. However, the function of *Drosophila* CRL4 during brain development is unknown.

Here, we show that the conserved E3 ubiquitin ligase CRL4 is essential for NSC reactivation in larval brains. Upon depletion of key components of CRL4, including the adaptor DDB1 and the scaffold Cul4, quiescent NSCs fail to reactivate. In addition, we have identified Mahjong (Mahj, *Drosophila* ortholog of human DCAF1) as a substrate receptor (DCAF) of CRL4 E3 during NSC reactivation: whereas NSC reactivation is compromised upon *mahj* depletion, Mahj overexpression is sufficient to promote premature NSC reactivation. We further show that CRL4^Mahj^ forms a protein complex with Wts, a key kinase of the Hippo pathway that maintains NSC quiescence. Furthermore, we demonstrate that Mahj, but not a truncated Mahj lacking its substrate-binding domain, promotes Wts ubiquitination. Moreover, knockdown of *wts* or overexpression of a constitutively active form of Yki, the transcription coactivator that is normally inhibited by active Wts, largely suppresses the quiescent NSC phenotypes caused by either *mahj*, *ddb1*, or *cul4* depletion. Thus, CRL4^Mahj^ E3 ubiquitinates and inhibits Wts, thereby activating Yki to trigger NSC reactivation.

## Results

### DDB1, the adaptor protein of CRL4 E3, is required for NSC reactivation

**Fig 1:**
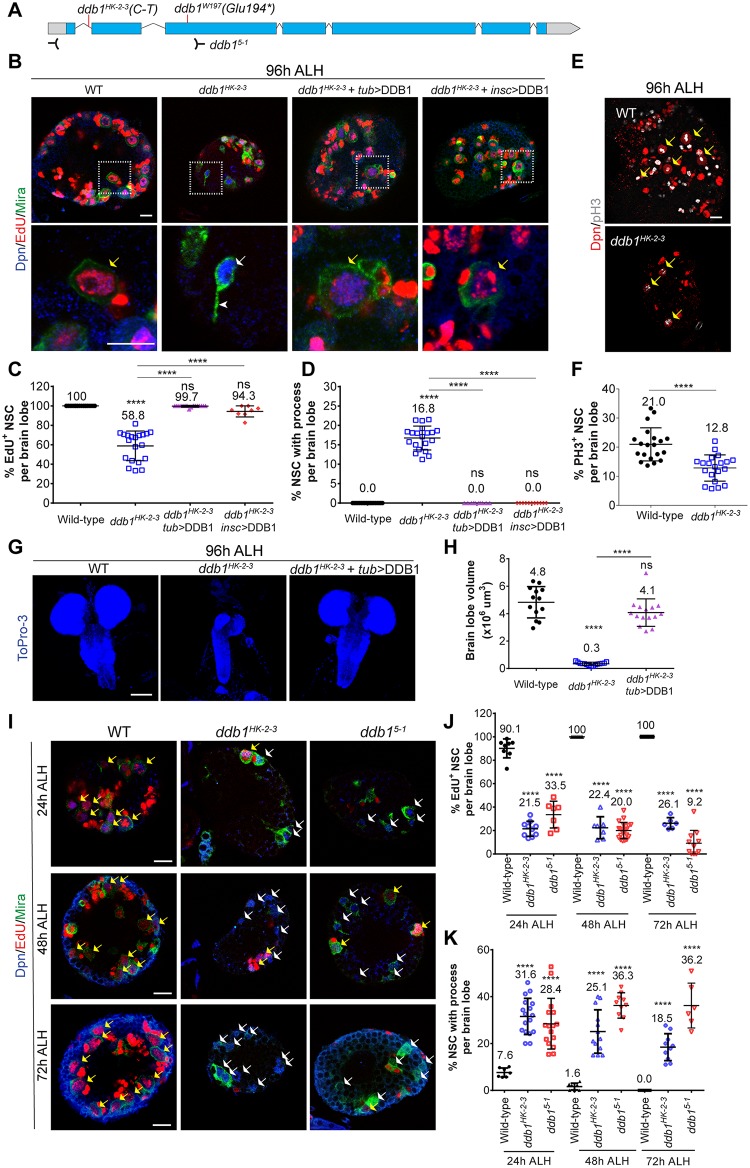
DDB1, the adaptor of CRL4, is required for NSC reactivation. (A) A schematic diagram showing the mutation/deletion sites in *ddb1* alleles. (B) At 96 h ALH, larval brain lobes from WT, *ddb1*^*HK-2-3*^ homozygous mutant, and *ddb1*^*HK-2-3*^ mutant expressing UAS-DDB1 under *tub*-Gal4 or *insc*-Gal4 were labeled with Dpn, EdU, and Mira. The lower panels are enlarged views of the white dotted boxes in the upper panels. (C-D) Quantification of Dpn^+^ Mira^+^ NSCs that are EdU^+^ (C) or display cellular process (D) in various genotypes. (E) PH3 and Dpn were labeled in WT and *ddb1*^*HK-2-3*^ larval brains. (F) Quantification of PH3^+^ Dpn^+^ NSCs of various genotypes in (E). (G) Maximum-intensity z-projection of larval brains from WT, *ddb1*^*HK-2-3*^, and *ddb1*^*HK-2-3*^ expressing *tub*>DDB1. (H) Quantification of brain lobe volume in (G). (I) EdU incorporation of NSCs in WT, *ddb1*^*HK-2-3*^, and *ddb1*^*5-1*^ at 24 h, 48 h, and 72 h ALH. (J-K) Quantification of Dpn^+^ Mira^+^ NSCs that are EdU^+^ (J) or that display cellular process (K) in various genotypes at indicated time points. For (J), at 48 h ALH, WT: *n* = 8 brain lobes, total NSCs t = 325; *ddb1*^*HK-2-3*^: *n* = 7, t = 200; *ddb1*^*5-1*^: *n* = 20, t = 713; At 72 h ALH, WT: *n* = 17, t = 672; *ddb1*^*HK-2-3*^: *n* = 6, t = 194; *ddb1*^*5-1*^: *n* = 11, t = 597. For (K), at 48 h ALH, WT: *n* = 8, t = 433; *ddb1*^*HK-2-3*^: *n* = 13, t = 766; *ddb1*^*5-1*^_:_ *n* = 10, t = 389. At 72 h ALH, WT: *n* = 21, t = 1,506; *ddb1*^*HK-2-3*^: *n* = 10, t = 855; *ddb1*^*5-1*^: *n* = 6, t = 236. Data are presented as mean ± SD. **** for *P* ≤ 0.0001. Yellow arrows, EdU^+^or PH3-positive NSCs. White arrows, NSCs without EdU or with a cellular process. Arrowheads, cellular process of quiescent NSCs. Scale bars: 10 μm (B, E, I) and 100 μm (G). *n*, number of brain lobes; t, total number of NSCs. The data underlying this figure can be found in S1 Data. ALH, after larval hatching; CRL4, Cullin4-RING ligase; DDB1, damaged DNA-binding protein 1; Dpn, Deadpan; EdU, 5-ethynyl-2′-deoxyuridine; Mira, Miranda; ns, statistically nonsignificant; NSC, neural stem cell; PH3, phospho-Histone H3; UAS, upstream activating sequence; WT, wild type.

To identify novel regulators of NSC proliferation, we carried out a genetic screen on a collection of chromosome 3R mutants induced by ethyl methane sulfonate (EMS) mutagenesis (refer to S1 Text). We isolated two new *ddb1* alleles that were henceforth named *ddb1*^*HK-2-3*^ and *ddb1*^*W197*^. DDB1 is a core component of CRL4 E3 ubiquitin ligase [20,21]. *ddb1*^*W197*^ contains a nonsense mutation (G to T at Glu194) likely resulting in a truncated protein, and *ddb1*^*HK-2-3*^ has a point mutation (C to T) at position −12 nucleotides from the splice acceptor site of the first intron (Fig 1A). To analyze NSC lineage development in these alleles, we generated mosaic analysis of repressible cell marker (MARCM) clones [33]. Whereas all control NSCs positive for Deadpan (Dpn), an NSC marker, were round in morphology (*n* = 63; *n* refers to total NSCs in MARCM clones), both *ddb1*^*W197*^ (14.6%, *n* = 48) and *ddb1*^*HK-2-3*^ (16.2%, *n* = 68) mutant NSCs showed a primary cellular process, which is a characteristic of quiescent NSCs [4,9] (S1A Fig). Consistent with a previous report that Miranda (Mira) is present in quiescent NSCs [7], cortical and cytoplasmic Mira were observed in all quiescent NSCs, including their cellular extensions. Therefore, we used a combination of nuclear Dpn and Mira^+^ cellular extension as markers for quiescent NSCs for most of our subsequent analysis. Consistently, whereas all control NSCs (*n* = 112) were positive for 5-ethynyl-2′-deoxyuridine (EdU), only 23.1% (*n* = 52) of *ddb1*^*W197*^ and 16.7% (*n* = 30) of *ddb1*^*HK-2-3*^ mutant NSCs were EdU^+^ (S1A Fig). NSC reactivation defects observed in *ddb1*^*W197*^ and *ddb1*^*HK-2-3*^ mutant MARCM clones were fully rescued by expressing upstream activating sequence (UAS)-*ddb1* (S1A Fig).

Since both *ddb1* alleles displayed similar phenotypes and the homozygous *ddb1*^*HK-2-3*^ survived to pupal stage, we henceforth focused our analysis of NSC reactivation using the *ddb1*^*HK-2-3*^ allele. All NSCs in wild-type (WT) larval brains that were positive for two NSC markers, Dpn and Mira, at 96 h ALH were round (*n* = 20, t = 1,677; *n* refers to number of brain lobes, and t refers to total NSCs) and incorporated EdU (*n* = 20, t = 1,340) (Fig 1B–1D). However, 16.8% (*n* = 20, t = 1,993) of NSCs in *ddb1*^*HK-2-3*^ extended a cellular process, and only 58.8% of NSCs (*n* = 20, t = 1,016) incorporated EdU (Fig 1B–1D). The quiescent NSC phenotype of *ddb1*^*HK-2-3*^ was well rescued by overexpression of UAS-*ddb1* driven by ubiquitous driver *tub*-Gal4 or NSC-specific driver *insc*-Gal4 (Fig 1B–1D). None of these *ddb1*^*HK-2-3*^ NSCs expressing DDB1 showed a cellular extension (*tub*-Gal4 driver, *n* = 10, t = 985; *insc*-Gal4 driver, *n* = 11, t = 1,084). In addition, 99.7% (*tub*-Gal4 driver, *n* = 18, t = 1,049) and 94.3% (*insc*-Gal4 driver, *n* = 8, t = 331) were positive for EdU (Fig 1B–1D). In addition, the number of mitotic cells that are positive for phospho-Histone H3 (PH3) was greatly reduced in the *ddb1*^*HK-2-3*^ mutant compared to that in WT (Fig 1E and 1F; WT: 21.0%, *n* = 20, t = 1,677; *ddb1*^*HK-2-3*^: 12.8%, *n* = 20, t = 1,660). At 96 h ALH, the small brain phenotype was largely rescued by overexpression of DDB1 driven by *tub*-Gal4 (Fig 1G and 1H; WT, 4.8 ± 1.1 × 10^6^ μm^3^, *n* = 13; *ddb1*^*HK-2-3*^, 0.3 ± 0.1 × 10^6^ μm^3^, *n* = 15; and *ddb1*^*HK-2-3*^ with *tub*>DDB1, 4.1 ± 1.0 × 10^6^ μm^3^, *n* = 15). The defects in NSC reactivation is a likely cause of the reduced central brain size in *ddb1*^*HK-2-3*^ compared with that in WT. As the entire brain lobe of *ddb1*^*HK-2-3*^ is smaller than that of the control, it is likely that DDB1 is also required for the development of the optic lobes. Moreover, we generated an anti-DDB1 antibody against the C terminus of DDB1. DDB1 consistently showed predominant nuclear staining in WT larval brains, which were also enriched in Mira^+^ NSCs (S1B Fig). By contrast, DDB1 was greatly diminished in *ddb1*^*HK-2-3*^ homozygous brains, especially in NSCs (S1B Fig), suggesting that *ddb1*^*HK-2-3*^ was a strong loss-of-function allele.

To further investigate the role of DDB1 during NSC reactivation, we carried out time-course experiments in *ddb1*^*HK-2-3*^ and a null allele, *ddb1*^*5-1*^ [24]. In WT larval brains at 24 h ALH, 90.1% (*n* = 8, t = 495) of NSCs incorporated EdU, and only 7.6% (*n* = 7, t = 463) of NSCs still retained the cellular process (Fig 1I–1K). By contrast, in *ddb1* mutants at 24 h ALH, only 21.5% (*ddb1*^*HK-2-3*^: *n* = 10, t = 183) and 33.5% (*ddb1*^*5-1*^: *n* = 7, t = 221) of NSCs incorporated EdU; and 31.6% (*ddb1*^*HK-2-3*^: *n* = 17, t = 1,008) and 28.4% (*ddb1*^*5-1*^: *n* = 16, t = 722) of NSCs still retained cellular process (Fig 1I–1K). Although at 48 h ALH and 72 h ALH almost all WT NSCs were reactivated, there were still significant numbers of NSCs from *ddb1*^*HK-2-3*^ and *ddb1*^*5-1*^ that remained in quiescence, as indicated by the lack of EdU incorporation and by the presence of the cellular process (Fig 1I–1K). Overall, the time-course experiment indicates that DDB1 is essential for NSCs to exit from quiescence.

Moreover, quiescent NSCs were observed upon knockdown of *ddb1 RNA interference (RNAi)* (Vienna Drosophila Resource Center [VDRC]#44974) driven by *insc*-Gal4 at 24 h ALH (S1C–S1F Fig), albeit with weaker phenotypes than in the *ddb1*^*HK-2-3*^ allele. The quiescent phenotypes could be fully rescued by overexpression of an RNAi-resistant UAS-*ddb1* construct expressed in NSCs at 96 h ALH (S1G–S1I Fig). At 96 h ALH, upon knocking down *ddb1* by a glial-driver *repo*-Gal4, no defects in NSC reactivation were observed (S1J–S1L Fig), further supporting the intrinsic requirement of DDB1 during NSC reactivation. At 0 h ALH, DDB1 was also detected in the nucleus of quiescent Dpn^+^ NSCs, but with similar intensity with surrounding cells (S1M Fig). This is different from strong DDB1 observed at 96 h ALH (S1B Fig), suggesting that DDB1 might be up-regulated in reactivated NSCs.

Taken together, these observations indicate that DDB1 is an intrinsic regulator of NSC reactivation.

### Cul4, the scaffold of CRL4 E3, and its ubiquitin ligase activity are required for NSC reactivation

**Fig 2:**
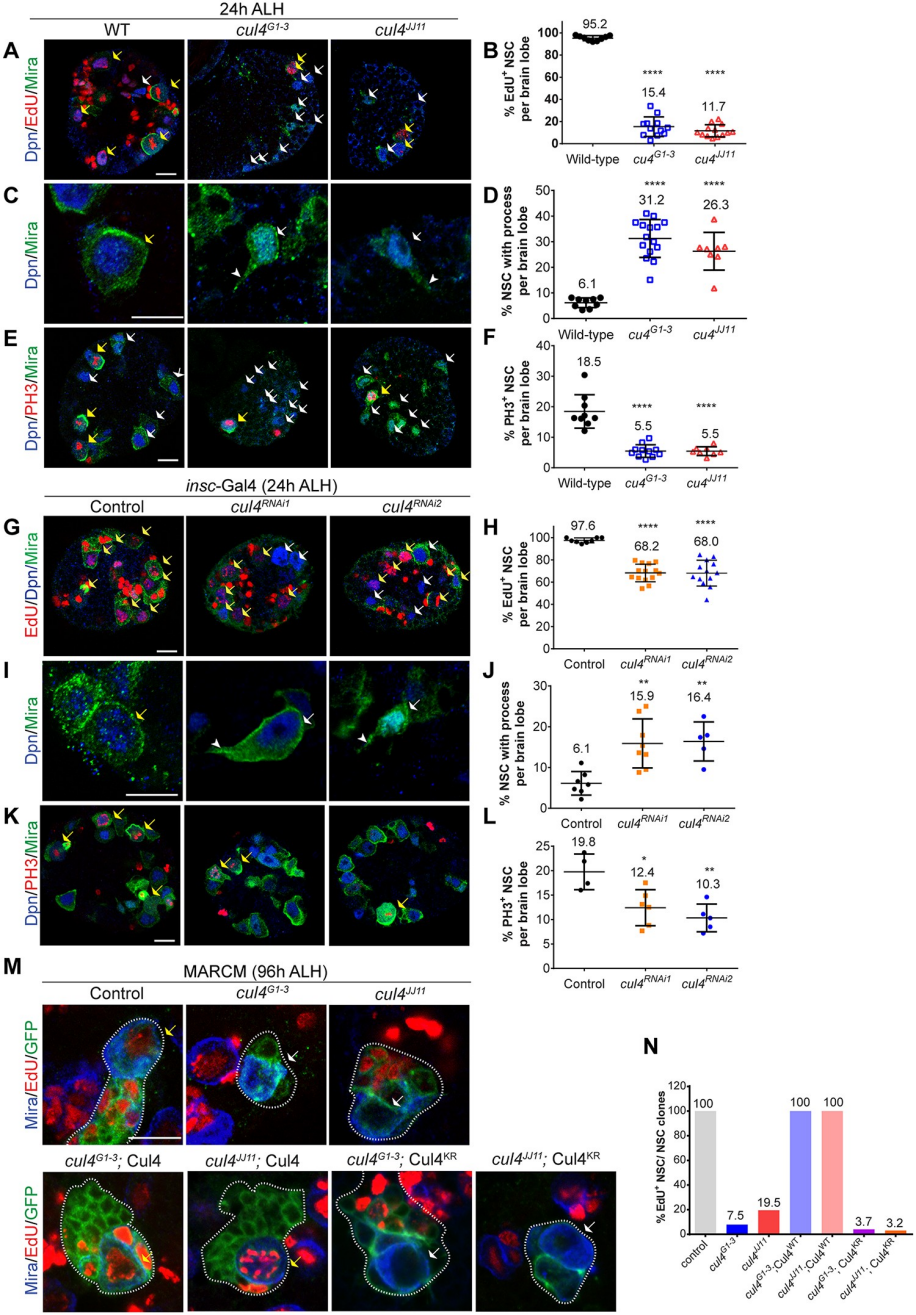
Cul4, the scaffold of CRL4, and its ubiquitin ligase activity are required for NSC reactivation. (A-F) Larval NSCs in WT, *cul4*^*G1-3*^, and *cul4*^*JJ11*^ homozygous mutants at 24 h ALH were labeled with Dpn, Mira, and EdU (A); Dpn and Mira (C); or Dpn, Mira, and PH3 (E). Quantifications in (A, C, E) were shown in (B), (D), and (F), respectively. (G-L) At 24 h ALH, larval brains in control (*β*-*gal*^RNAi^), *cul4*^RNAi1^ (VDRC#105668), and *cul4*^RNAi2^ (VDRC#44829) under *insc*-Gal4; UAS-Dcr2 were labeled with Dpn, Mira, and EdU (G); Dpn and Mira (I); or Dpn, Mira, and PH3 (K). Quantifications in (G, I, K) were shown in (H), (J), and (L), respectively. In (H), control: *n* = 8 brain lobes, total NSCs t = 405; *cul4*^RNAi1^, *n* = 14, t = 606; *cul4*^RNAi2^: *n* = 13, t = 538. In (J), control: *n* = 7, t = 562; *cul4*^RNAi1^, *n* = 8, t = 530; *cul4*^RNAi2^: *n* = 5, t = 290. Data are presented as mean ± SD. **** for *P* ≤ 0.0001, ** for *P* ≤ 0.01. (M) MARCM clones of control (FRT42D), *cul4*^*G1-3*^, *cul4*^*JJ11*^, UAS-Flag-Cul4^WT^; *cul4*^*G1-3*^, UAS-Flag-Cul4^WT^; *cul4*^*JJ11*^, UAS-Flag-Cul4^KR^; *cul4*^*G1-3*^, and UAS-Flag-Cul4^KR^; *cul4*^*JJ11*^ were labeled with Mira, EdU, and CD8-GFP. (N) The percentage of EdU^+^ Mira^+^ NSCs of indicated genotypes in (M). White dotted lines mark MARCM clones labeled by CD8-GFP. Yellow arrows, EdU^+^ or PH3^+^ NSCs. White arrows, EdU-negative or process-retaining NSCs. Arrowheads, cellular process of quiescent NSCs. Scale bar: 10 μm. *n*, number of brain lobes; t, total number of NSCs. The data underlying this figure can be found in S1 Data. ALH, after larval hatching; CRL4, Cullin4-RING ligase; Cul4, Cullin4; Dcr2, Dicer 2; Dpn, Deadpan; EdU, 5-ethynyl-2′-deoxyuridine; GFP, green fluorescent protein; MARCM, mosaic analysis of repressible cell marker; Mira, Miranda; NSC, neural stem cell; PH3, phospho-Histone H3; UAS, upstream activating sequence; VDRC, Vienna Drosophila Resource Center; WT, wild type.

Scaffold Cul4 is another core component of the CRL4 E3, and it directly binds to DDB1 [20,21]. To investigate the function of Cul4 during NSC reactivation, we examined homozygous mutant brains of *cul4*^*G1-3*^ and *cul4*^*JJ11*^, two null alleles of *cul4* [24]. At 24 h ALH, there were significantly fewer EdU^+^ NSCs in *cul4*^*G1-3*^ and *cul4*^*JJ11*^ mutants compared with stage-matched WT (Fig 2A and 2B; WT, 95.2%, n = 9, t = 437; *cul4*^*G1-3*^, 15.4%, *n* = 12, t = 429; *cul4*^*JJ11*^, 11.7%, *n* = 13, t = 502). Similarly, 31.2% (*n* = 16, t = 694) and 26.3% (*n* = 8, t = 543) of NSCs extended a cellular process in *cul4*^*G1-3*^ and *cul4*^*JJ11*^ mutants, compared with only 6.1% of NSCs (*n* = 9, t = 543) of WT brain lobes (Fig 2C and 2D). The proportion of mitotic NSCs marked by PH3 was also significantly decreased in *cul4*^*G1-3*^ and *cul4*^*JJ11*^ mutants compared with WT (Fig 2E and 2F; WT, 18.5%, *n* = 9, t = 620; *cul4*^*G1-3*^, 5.5%, *n* = 11, t = 566; *cul4*^*JJ11*^, 5.5%, *n* = 8, t = 543). Moreover, at 24 h ALH upon knockdown of *cul4* by two different RNAi lines driven by NSC-driver *insc*-Gal4, significantly more quiescent NSCs, as judged by the lack of EdU incorporation and by the presence of cellular processes, were observed (Fig 2G–2J). For *cul4* RNAi, based on similar phenotypes observed with *cul4* mutants, it is most likely that *cul4* RNAi knockdown is efficient. Furthermore, the population of mitotic PH3^+^ NSCs was also significantly reduced upon *cul4* knockdown: 19.8% PH3^+^ NSCs in the control at 24 h ALH (*n* = 4, t = 316) versus 12.4% (*cul4*^RNAi1^, *n* = 6, t = 399) and 10.3% (*cul4*^RNAi2^, *n* = 5, t = 290) PH3^+^ NSCs were observed in *cul4*^RNAi1^/VDRC#105668 and *cul4*^RNAi2^/VDRC#44829 (Fig 2K and 2L). Furthermore, knockdown of *cul4* by RNAi under *repo*-Gal4 at 96 h ALH did not cause significant defects in NSC reactivation (S2A–S2C Fig). These results indicate that scaffold protein Cul4 of the CRL4 complex is also an intrinsic regulator of NSC reactivation. A group of four mushroom body (MB) NSCs in larval central brains divide throughout the larval stages independently of dietary amino acids and thus do not enter or exit from quiescence [6]. All MB NSCs in control brains (*n* = 26, t = 104) and *ddb1*^*HK-2-3*^ (*n* = 21, t = 84), *ddb1*^*5-1*^ (*n* = 20, t = 80), and *cul4*^*G1-3*^ mutant brains (*n* = 23, t = 92) were proliferating at 24 h ALH on food depleted of amino acids, as indicated by their ability to incorporate EdU (S2H Fig). We conclude that loss of *ddb1* or *cul4* does not obviously affect MB NSC proliferation.

We wondered whether Cul4 regulates NSC reactivation through its function as part of the CRL4 E3 ligase. Neddylation, a reversible attachment of the ubiquitin-like protein NEDD8 to Cullins, is essential for the enzymatic activity of the CRL family of E3 ligases [34,35]. We took advantage of a dominant-negative form of Cul4 named Cul4^KR^, in which the neddylation site has been mutated [24]. Whereas overexpression of wild-type Cul4^WT^ driven by *insc*-Gal4 did not result in a significant change of EdU^+^ NSCs compared with that of control brains (control: 97.9%, *n* = 8, *t* = 422; Cul4^WT^: 96.4%, *n* = 16, *t* = 1,076), overexpression of Cul4^KR^ resulted in a marked decrease of EdU^+^ NSCs to 57.2% (*n* = 11, *t* = 567) (S2D and S2E Fig). Furthermore, significantly more NSCs with a cellular process and fewer PH3-positive NSCs were observed in larval brains upon overexpression of Cul4^KR^ than of Cul4^WT^ or control (S2F and S2G Fig). These phenotypes suggest that Cul4^KR^ overexpression also generates a dominant-negative effect during NSC reactivation. *cul4*^*G1-3*^ and *cul4*^*JJ11*^ MARCM clones displayed similar NSC proliferation defects as homozygous *cul4*^*G1-3*^ and *cul4*^*JJ11*^ mutants (Fig 2M and 2N). At 96 h ALH, whereas all Mira^+^ NSCs (*n* = 65) in control MARCM clones were EdU^+^, only 7.5% (*n* = 53) and 19.5% (*n* = 41) of Mira^+^ NSCs in *cul4*^*G1-3*^ and *cul4*^*JJ11*^ MARCM clones were EdU^+^ (Fig 2M and 2N). The quiescent NSC phenotype of *cul4*^*G1-3*^ and *cul4*^*JJ11*^ was completely rescued by overexpression of UAS-*cul4* transgene (Fig 2M and 2N; *n* = 105 and *n* = 69). By contrast, overexpression of UAS-*Cul4*^*KR*^ failed to rescue the quiescent NSC phenotypes of *cul4*^*G1-3*^ and *cul4*^*JJ11*^, as indicated by 3.7% (*n* = 27) and 3.2% (*n* = 31) EdU^+^ NSCs, respectively (Fig 2M and 2N). These results suggested that Cul4 neddylation and, in turn, the ubiquitin ligase activity of CRL4 E3 are essential for NSC proliferation.

### DDB1 likely functions downstream of InR pathway during NSC reactivation

We next tested whether DDB1 is required for premature reactivation induced by overactivation of InR signaling pathway. Similar to previous reports [10,11], in the absence of dietary amino acids, control larval brains contained on average 3.9 EdU^+^ NSCs (S2I and S2J Fig; *n* = 13 brain lobes, t = 51 NSCs), whereas overexpression of an active form of InR (InR^CA^) in NSCs triggered NSC reactivation (S2I and S2J Fig; 34.2 EdU^+^ NSCs per brain lobe, *n* = 10, t = 342). By contrast, *ddb1* knockdown dramatically suppressed this effect induced by InR^CA^ overexpression (S2I and S2J Fig; 6.2 EdU^+^ NSCs per brain lobe, *n* = 16, t = 99). This result suggested that DDB1 likely functions downstream of the InR pathway during NSC reactivation.

### Mahj, a potential substrate receptor of CRL4 E3 ligase, is required for NSCs to exit from quiescence

Most substrate receptors of CRL4, referred to as DCAFs, contain one or more WDXR motifs in their WD40 domain [22,23]. To identify specific DCAFs of CRL4 that regulate NSC reactivation, we screened a collection of RNAi lines that individually knock down each of 44 genes encoding potential DCAF proteins for their effects during NSC reactivation (Table 1). From this screen, we identified Mahj (encoded by CG10080), the *Drosophila* ortholog of human DCAF1/Vpr (HIV-1) Binding Protein (VprBP) [36], as the only potential regulator of NSC reactivation among those 44 candidate genes. At 24 h ALH, whereas only 3.7% (*n* = 9, t = 649) of NSCs in control brain lobes displayed a cellular process, knockdown of *mahj* by two different RNA lines (*mahj*^RNAi1^: Bloomington *Drosophila* Stock Center [BDSC]#34912 and *mahj*^RNAi2^: VDRC#110669) driven by *insc*-Gal4 led to an increase in proportion of NSCs with a cellular process to 23.1% (*mahj*^RNAi1^, *n* = 16, t = 1,242) and 27.1% (*mahj*^RNAi2^, *n* = 7, t = 575) (S3A and S3B Fig). Consistently, EdU incorporation by NSCs was reduced to 63.3% (*n* = 8, t = 478) and 75.1% (*n* = 16, *t* = 834) upon *mahj* RNAi knockdown, whereas there were 96.6% (*n* = 7, t = 377) EdU^+^ NSCs in the control (S3C and S3D Fig). In addition, the percentage of mitotic PH3^+^ NSCs was also reduced significantly to 7.2% (*n* = 7, t = 500) in *mahj*^RNAi1^ and 9.1% (*n* = 7, t = 575) in *mahj*^RNAi2^, compared with 20.6% (*n* = 6, t = 444) in control (S3E and S3F Fig).

**Table 1:** Related to Fig 3. *Drosophila* DCAFs and their RNAi lines.

S/No	Gene name	CG	RNAi lines
1	CG3436	CG3436	103140KK
2	CG3515	CG3515	106711KK
3	CG10646	CG10646	100744KK
4	Mahjong	CG10080	34912TriP 110699KK
5	DDA1	CG31855	108090KK 34295GD
6	CG14614	CG14614	107076KK
7	Yippee	CG1989	108836 KK
8	Ohgt	CG3925	110809KK
9	CG15309	CG15309	101370KK
10	CG7568	CG7568	110369 KK
11	CG7275	CG7275	103948KK
12	CG8001	CG8001	35318GD
13	Nup43	CG7671	108595KK
14	WMD	CG3957	106171KK
15	CG10064	CG10064	106172KK
16	slimb	CG3412	107825KK
17	abo	CG6093	105384KK
18	bchs	CG14941	110785KK
19	esc	CG14941	5692GD
20	Rae1	CG9862	101338KK
21	CG1523	CG1523	110437KK
22	CG10931	CG10931	51704GD
23	CG9945	CG9945	105944KK
24	CG12134	CG12134	31654GD
25	Lethal (2) 09851	CG12792	110333KK
26	Wdr24	CG7609	108562KK
27	WDA	CG4448	34847GD
28	CG4705	CG4705	108007KK
29	WDR82	CG17293	25246GD
30	Oseg6	CG11237	38462GD
31	Caf1-105	CG12892	110461KK
32	Atg16	CG31033	105993KK
33	CG1671	CG1671	105679KK
34	U4-U6-60K	CG6322	110393KK
35	CG14353	CG14353	105833KK
36	Groucho	CG8384	110546KK
37	CG1571	CG1571	51846GD
38	Elp2	CG11887	105393KK
39	Prp19	CG5519	108575KK
40	CG5543	CG5543	106320GD
41	Caf1-55	CG4236	105838KK
42	Fzr	CG3000	25553GD
43	Aladin	CG16892	101415KK
44	Poc1	CG10191	108219KK

Abbreviations: abo, abnormal oocyte; Atg16, Autophagy-related 16; bchs, blue cheese; Caf1-105, Chromatin assembly factor 1, p105 subunit; Caf1-55, Chromatin assembly factor 1, p55 subunit; DCAF, DDB1-Cul4 associated factor; DDA1, DET1- and DDB1-associated protein 1; Elp2, Elongator complex protein 2; esc, extra sexcombs; Fzr, fizzy-related; Nup43, Nucleoporin 43kD; Ohgt, Ohgata; Oseg6, Outer segment 6; Poc1, Proteome of centrioles 1; Prp19, Pre-mRNA processing 19; RNAi, RNA interference; slimb, supernumerary limbs; U4-U6-60K, U4-U6 small nuclear riboprotein factor 60K; WDA, Will Decrease Acetylation; Wdr, WD repeat domain; WMD, Wing Morphogenesis Defect.

**Fig 3:**
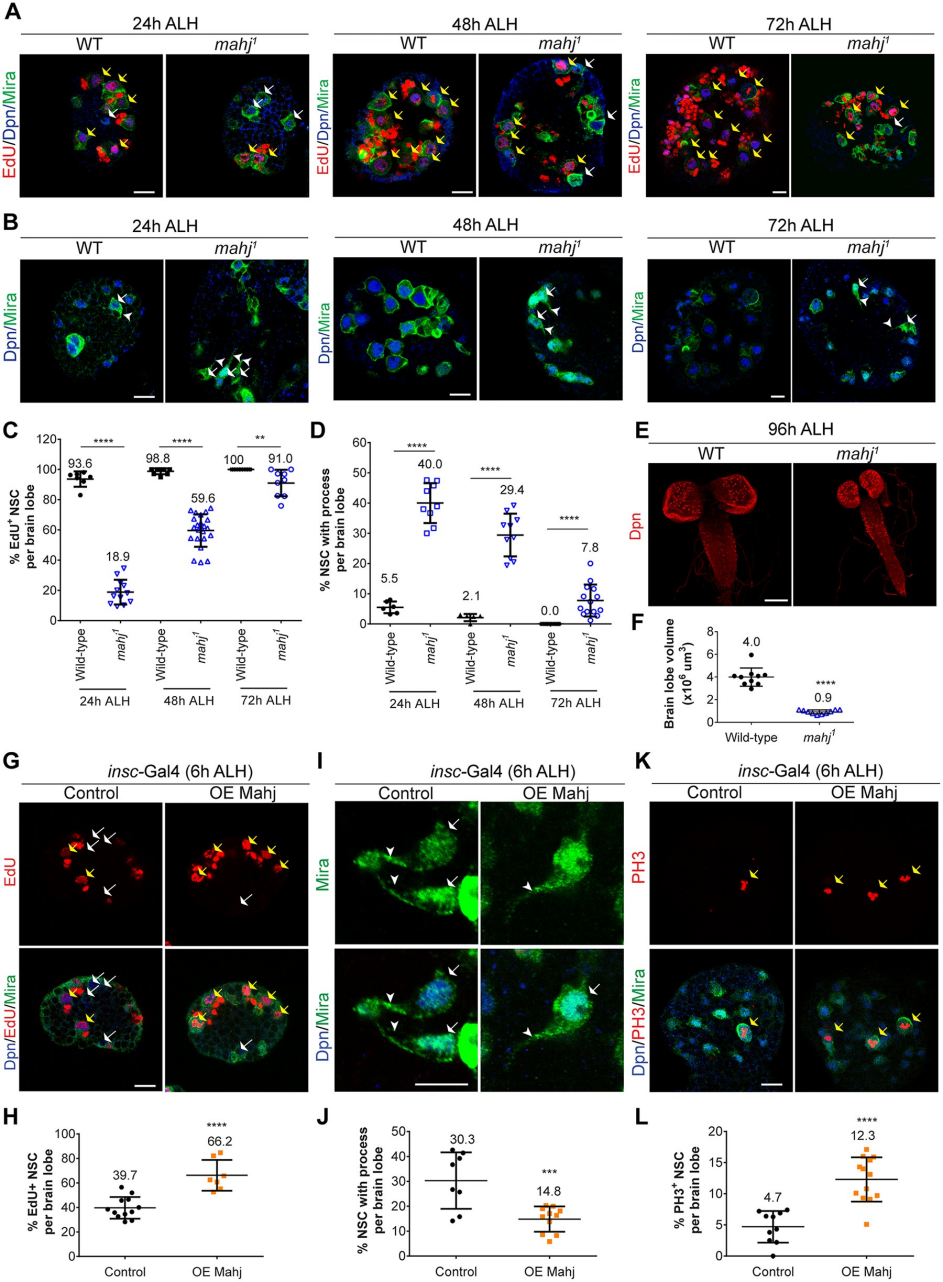
Mahj, a potential substrate receptor of CRL4, is required for NSC reactivation. (A-B) Larval NSCs in WT and *mahj*^*1*^ homozygous mutants at 24 h, 48 h, and 72 h ALH were labeled with EdU, Dpn, and Mira (A) or Dpn and Mira (B). (C-D) Quantification of Dpn^+^ Mira^+^ NSCs that are EdU^+^ (C) or display cellular process (D). (E) Maximum-intensity z-projection of larval brains from WT and *mahj*^*1*^ at 96 h ALH. NSCs were labeled with Dpn. (F) Quantification of brain lobe volume in (E). (G-L) Larval brain lobes from control (*β*-*gal*^RNAi^) and UAS-*mahj* under *insc*-Gal4; UAS-Dcr2 driver at 6 h ALH. NSCs were labeled with Dpn, EdU, and Mira in (G) or Dpn and Mira (I) or Dpn, PH3, and Mira (K). Quantification of NSCs that are EdU^+^ or with cellular process or positive for mitotic marker PH3 in (G, I, K) was shown in (H), (J), and (L). Data are presented as mean ± SD. **** for *P* ≤ 0.0001, *** for *P* ≤ 0.001, ** for *P* ≤ 0.01. Yellow arrows, EdU^+^ or PH3-positive NSCs. White arrows, NSCs without EdU or with a cellular process. Arrowheads, cellular process of quiescent NSCs. Scale bars, 10 μm (A-B, G-K) and 100 μm (E). The data underlying this figure can be found in S1 Data. ALH, after larval hatching; CRL4, Cullin4-RING ligase; Dcr2, Dicer 2; Dpn, Deadpan; EdU, 5-ethynyl-2′-deoxyuridine; Mahj, Mahjong; Mira, Miranda; NSC, neural stem cell; OE, overexpression; PH3, phospho-Histone H3; UAS, upstream activating sequence; WT, wild type.

To further investigate the roles of Mahj in NSC reactivation, we examined a loss-of-function allele, *mahj*^*1*^, that was previously generated by imprecise P-element excision [36]. In a time-course experiment, at 24 h ALH 93.6% (*n* = 7, t = 238) of WT NSCs incorporated EdU (Fig 3A and 3C). However, only 18.9% (*n* = 12, t = 401) of *mahj*^*1*^ NSCs were EdU^+^ (Fig 3A and 3C). At 48 h ALH, whereas 98.8% (*n* = 9, t = 310) of WT NSCs were EdU^+^, only 59.6% (*n* = 22, t = 748) of *mahj*^*1*^ NSCs incorporated EdU (Fig 3A and 3C). By 72 h ALH, most NSCs of *mahj*^*1*^ NSCs eventually became EdU^+^ (Fig 3A and 3C; *mahj*^*1*^, 91.0%, *n* = 9, t = 485 compared with WT, 100%, *n* = 13, t = 437). Next, we examined the cellular extension in *mahj*^*1*^ quiescent NSCs. At 24 h ALH and 48 h ALH, 5.5% (*n* = 6, t = 251) and 2.1% (*n* = 5, t = 186) of WT NSCs displayed cellular extension(s), and none of them retained any cellular extension by 72 h ALH (*n* = 18, t = 617) (Fig 3B and 3D). By contrast, in *mahj*^*1*^ brains there was a much higher proportion of Dpn^+^ Mira^+^ NSCs that retained cellular processes: 40.0% (*n* = 9, t = 401) at 24 h ALH, 29.4% (*n* = 10, t = 486) at 48 h ALH, and 7.8% (*n* = 14, t = 1,013) at 72 h ALH, respectively (Fig 3B and 3D). We generated a specific anti-Mahj antibody and found that Mahj was nuclear in most WT brain cells and was enriched in NSCs (S3G Fig). The localization pattern of Mahj in the larval brain was similar to that of DDB1. Mahj was undetectable in *mahj*^*1*^ mutant brains by immunostaining (S3G Fig). Consistently, a cross-reacting Mahj protein of the expected size (approximately 180 kDa) was observed in 24 h ALH lysates of WT but not in those of *mahj*^*1*^ (S3H and S3I Fig). Like *ddb1*^*HK-2-3*^, *mahj*^*1*^ brain lobes at 96 h ALH were significantly smaller than those of WT (Fig 3E and 3F, 0.9 ± 0.2 × 10^6^ μm^3^, *n* = 10 for *mahj*^*1*^ versus 4.0 ± 0.81 × 10^6^ μm^3^, *n* = 10 for WT). Whereas only 58.6% of *mahj*^*1*^ NSCs (*n* = 10, t = 541) were EdU^+^at 48 h ALH, 90.6% (*n* = 10 brain lobes, t = 735) of *mahj*^*1*^ NSCs expressing UAS-Mahj under *insc*-Gal4 incorporated EdU (S3J and S3K Fig), suggesting that the quiescent NSC phenotype of *mahj*^*1*^ was essentially rescued by overexpression of Mahj. Moreover, the transheterozygous mutants of *mahj*^*1*^*/Df* also displayed NSC reactivation defects, as judged by the reduction of EdU^+^ NSCs (S3L and S3M Fig; WT: 93.3%, *n* = 10, t = 733 versus *mahj*^*1*^*/Df*: 12.8%, *n* = 12, t = 610) and the increase in percentage of NSCs with processes (S3L and S3N Fig; WT: 6.7%, *n* = 4, t = 366 versus *mahj*^*1*^*/Df*: 30.7%, *n* = 6, t = 333).

MB NSCs in both WT brains (*n* = 22, t = 88) and *mahj*^*1*^ brains (*n* = 16, t = 64) were proliferating at 24 h ALH, as indicated by their ability to incorporate EdU (S3O Fig). Although we cannot formally exclude the possibility that Mahj plays a minor role in proliferation of MB NSCs, our current data suggest that *mahj* depletion significantly delays NSC reactivation without impeding general cell proliferation. Next, we examined whether Mahj protein is present in quiescent NSCs. At 0 h ALH, Mahj can be seen in the nucleus of quiescent Dpn^+^ NSCs in larval brains (S3P Fig).

The number of NSCs in *ddb1*^−^, *cul4*^−^, and *mahj*^−^ mutant brains at 24 h ALH were significantly reduced (*ddb1*^*HK-2-3*^, 54.2 ± 4 NSCs per brain lobe, *n* = 10; *cul4*^*JJ11*^, 33.3 ± 6.3, *n* = 9; *mahj*^*1*^, 53.9 ± 4.7, *n* = 11; S3Q Fig), possibly because of defects in neurogenesis at the embryonic stages. This reduction of NSC numbers likely in part contributed to the reduced brain volume observed in *ddb1*^*HK-2-3*^ and *mahj*^*1*^. Although we cannot exclude the possibility that NSC reactivation defects are partially due to reduced NSC numbers and that reactivation of a specific class of NSCs is affected in *ddb1*^−^, *cul4*^−^, and *mahj*^−^ mutants, the severe EdU incorporation defects in these mutants cannot be solely explained by reduced NSC numbers.

### Mahj overexpression leads to premature reactivation of NSCs

Since loss of *mahj* resulted in delayed NSC reactivation, we wondered whether overexpression of Mahj would be sufficient to induce premature NSC reactivation. At 6 h ALH, overexpression of full-length Mahj (UAS-*Mahj*) driven by *insc*-Gal4 resulted in significantly more Dpn^+^ Mira^+^ NSCs with EdU incorporation than in the control: 66.2% (*n* = 7, t = 312) of NSCs with Mahj overexpression were EdU^+^ compared with 39.7% (*n* = 12, t = 574) in control brains (Fig 3G and 3H). In addition, at 6 h ALH, whereas 30.3% of control NSCs (*n* = 8, t = 534) displayed a cellular process, only 14.8% of Mahj-overexpressing NSCs (*n* = 11, t = 753) retained a cellular extension (Fig 3I and 3J). Moreover, Mahj overexpression resulted in an increased population of mitotic NSCs marked by PH3: 12.3% (*n* = 13, t = 899) PH3^+^ NSCs upon Mahj overexpression compared with 4.7% (*n* = 10, t = 683) in the control (Fig 3K and 3L). Taken together, these observations indicate that Mahj is both necessary and sufficient for NSC reactivation in the presence of nutrition.

### Mahj forms a protein complex with DDB1 and Cul4

**Fig 4:**
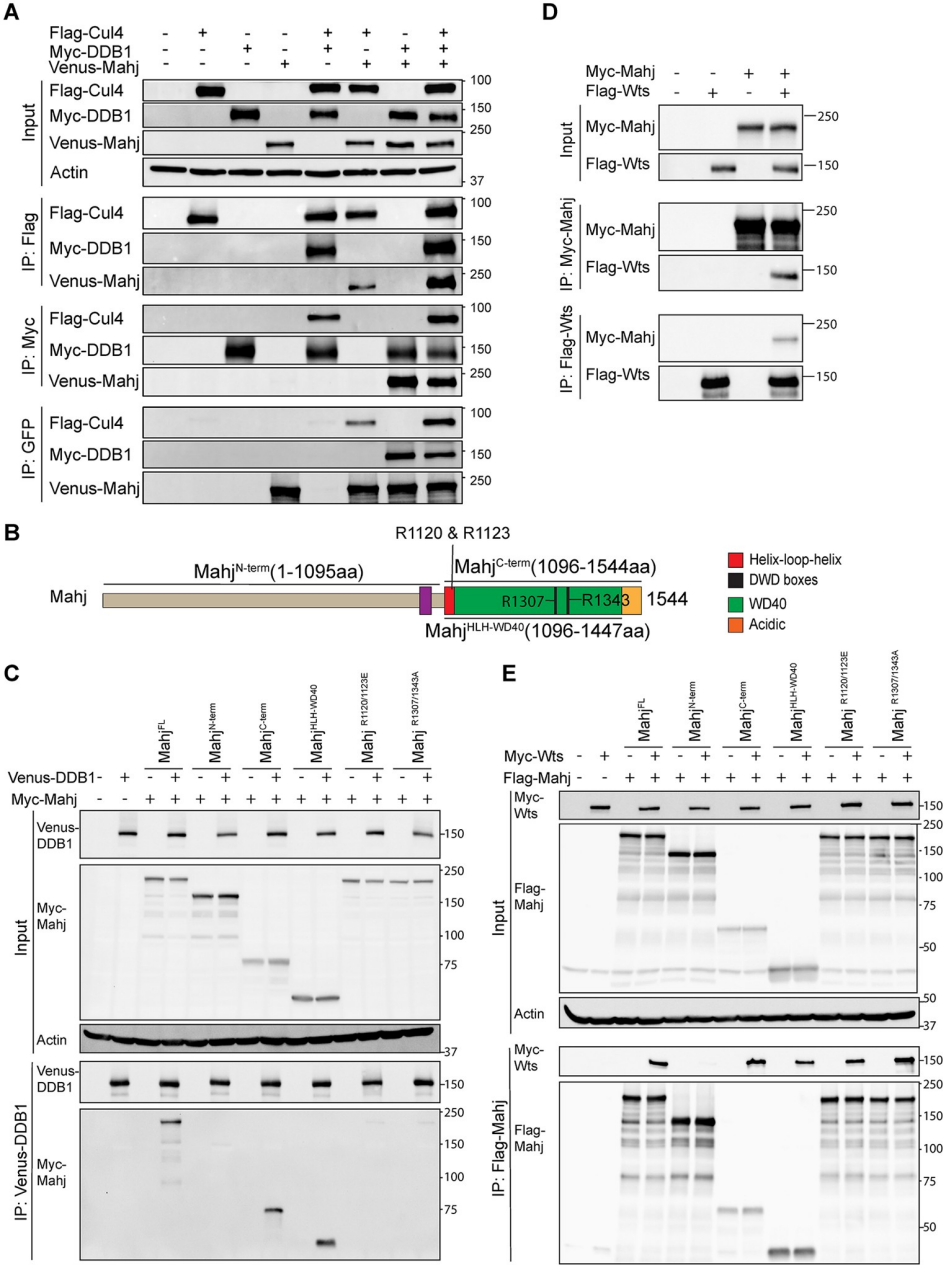
Mahj forms a protein complex with DDB1, Cul4, and Wts. (A) Co-IP among Flag-Cul4, Myc-DDB1, and Venus-Mahj. (B) A schematic diagram showing Mahj domains and mutated constructs. Mahj R1120 and R1123 are corresponding to human DCAF1 R1053 and R1056; Mahj R1307 and R1343 are corresponding to human DCAF1 R1247 and R1283. (C) Co-IP between Venus-DDB1 and FL, truncated or mutated forms of Myc-Mahj. (D) Co-IP between Myc-Mahj and Flag-Wts. (E) Co-IP between Myc-Wts and FL, truncated or mutated forms of Flag-Mahj. Actin served as a loading control. aa, amino acid; Cul4, Cullin4; DCAF, DDB1-Cul4 associated factor; DDB1, damaged DNA-binding protein 1; DWD, DDB1-binding WD40; FL, full-length; GFP, green fluorescent protein; IP, immunoprecipitation; Mahj, Mahjong; Wts, Warts.

It was unknown whether *Drosophila* Mahj physically associates with the CRL4 complex. To determine this, we cotransfected Flag-Cul4, Myc-DDB1, and Venus-Mahj into S2 cells and carried out coimmunoprecipitation (co-IP) experiments. Indeed, Venus-Mahj was detected in the same immune complex following immunoprecipitation (IP) of either Flag-Cul4 or Myc-DDB1 (Fig 4A). Significantly more Venus-Mahj was detected following IP of Flag-Cul4 when Myc-DDB1 was overexpressed (Fig 4A, compare lane 8 with lane 6 in IP-Flag). This observation suggested that Mahj, as a substrate receptor, likely associates with Cul4 through the adaptor protein DDB1. Consistently, Myc-DDB1 and Flag-Cul4 were detected in the immune complex following the IP of Venus-Mahj (Fig 4A). Again, the association between Flag-Cul4 and Venus-Mahj were strongly enhanced by the overexpression of Myc-DDB1 (Fig 4A, compare lane 8 with lane 6 in IP-green fluorescent protein [GFP]). We conclude that Mahj, DDB1, and Cul4 form a protein complex in which DDB1 serves as the linker between Cul4 and Mahj.

Comparing with human (*Homo sapiens* [*Hs*]) DCAF1, *Drosophila melanogaster* (*Dm*) Mahj also contains a LisH motif, a helix-loop-helix (HLH) motif, a WD40 domain, and an acidic domain (Fig 4B). The LisH motif of *Hs*DCAF1 is responsible for the dimerization of DCAF1 [37], whereas the HLH motif and WD40 domain are important for the binding of DCAF1 to DDB1 [38–40]. From our homology model of the *Dm*Mahj-*Dm*DDB1 complex, which was generated from the published crystal structure of the *Hs*DCAF1-*Hs*DDB1 complex (Protein Data Bank [PDB] 5JK7 [39]), Mahj likely uses its HLH motif and WD40 domain to interact with DDB1 (S4A–S4C Fig). To test if HLH motif and WD40 domain are indeed essential for Mahj interaction with DDB1, we generated various truncated forms of Mahj (Fig 4B) and tested their ability to physically associate with DDB1 in co-IP experiments. Like full-length Myc-tagged Mahj (Mahj^FL^), Myc-Mahj^C-term^ (1,096–1,544 aa, containing the HLH motif, WD40 domain, and acidic region) and Myc-Mahj^HLH+WD40^ (1,096–1,447 aa, containing the HLH motif and WD40 domain) were able to associate with Venus-DDB1 in co-IPs (Fig 4C). By contrast, the association between Venus-DDB1 and Myc-Mahj^N-term^ that lacked the HLH motif and WD40 domain was strongly diminished (Fig 4C). These results indicate that the HLH motif and WD40 domain of Mahj are critical for Mahj interaction with DDB1.

In *Hs*DCAF1, R1053 and R1507 in the HLH motif and R1247 and R1283 in the WD40 domain are critical for binding to *Hs*DDB1 [38,39]. We generated two mutant forms of Mahj at the sites of conserved arginine residues (R1120/R1123 and R1307/R1343, based on our alignment of Mahj with DCAF1 and a homology model of Mahj complexed with DDB1) by mutating the residues to glutamate (R1120/1123E) or alanine (R1307/1343A) (the sites of mutation are shown in Fig 4B and S4A–S4C Fig). The mutants were tested for their ability to associate with DDB1. As expected, in the co-IP experiments in S2 cells, both pairs of mutated residues strongly reduced the interactions between DDB1 and Mahj (Fig 4C). The disruption of these interactions was unlikely to be due to a major conformational change of the mutant proteins, as the mutated residues were located at the interaction surface between DDB1 and Mahj in the homology model (S4A–S4C Fig). Overexpression of the DDB1-binding defective mutant Mahj^R1120/1123E^ failed to rescue the quiescent NSC phenotypes of *mahj*^*1*^, as indicated by 66.4% (*n* = 12, t = 791) EdU^+^ NSCs versus 58.6% in *mahj*^*1*^ NSCs (*n* = 10, t = 541), unlike that of the WT Mahj (S3J and S3K Fig). Taken together, these results suggest that Mahj functions in a complex with DDB1 through the HLH motif and WD40 domain to regulate NSC reactivation.

### The substrate receptor Mahj physically associates with Wts

Given that CRL4^Mahj^ stimulates NSC reactivation and that CRL4^Mahj^ usually promotes ubiquitination and proteasome-mediated degradation of its substrates, we reasoned that substrates of CRL4^Mahj^ likely maintain NSC quiescence. Recently, Hippo pathway was shown to maintain NSC quiescence, and the loss of Hippo pathway core kinases such as Wts (*Drosophila* ortholog of human large tumor suppressor 1/2 [LATS1/2]) resulted in premature NSC reactivation [17,18]. This prompted us to test whether CRL4^Mahj^ ubiquitinates and inhibits Wts during NSC reactivation. If this is true, Wts needs to be recruited to CRL4^Mahj^. Indeed, Flag-Wts was detected in the immune complex when Myc-Mahj was immunoprecipitated from S2 cells (Fig 4D). Consistently, Myc-Mahj was also specifically detected in the immune complex with Flag-Wts in a reciprocal co-IP (Fig 4D). These results indicate that Mahj can interact with Wts in vitro.

Besides binding to *Hs*DDB1, the WD40 domain of *Hs*DCAF1 is also critical for interaction with substrates [38,40]. Therefore, we explored whether the WD40 domain of Mahj mediated its interaction with Wts. Indeed, whereas Flag-Mahj^FL^, Flag-Mahj^C-term^, and Flag-Mahj^HLH+WD40^ still associated with Myc-Wts, the interaction between Myc-Wts and Flag-Mahj^N-term^ lacking the WD40 domain (and HLH motif) was strongly diminished (Fig 4E). These observations suggest that the WD40 domain of Mahj is likely required for its physical association with Wts. As expected, two mutants (Mahj^R1120/1123E^ and Mahj^R1307/R1343A^), which were designed to disrupt DDB1-binding site specifically but not the substrate-binding site in Mahj, did not attenuate Mahj-Wts interaction (Fig 4E). These results suggest that the HLH motif and WD40 domain of Mahj represent the minimum fragment to interact with both DDB1 and Wts.

To investigate which domain of Wts mediates its association with Mahj, we generated various truncated forms of Wts (S4D Fig) and tested their interaction with Mahj by co-IP. Similar to Flag-Wts^FL^, Flag-Wts^C-term^ and Flag-Wts^Kinase^ were associated with Myc-Mahj, whereas Flag-Wts^N-term^, which lacked its kinase domain, did not interact with Myc-Mahj (S4E Fig). These results indicate that the C-terminal fragment containing the kinase domain of Wts is critical for its interaction with Mahj.

### Wts physically associates with DDB1 and Cul4

**Fig 5:**
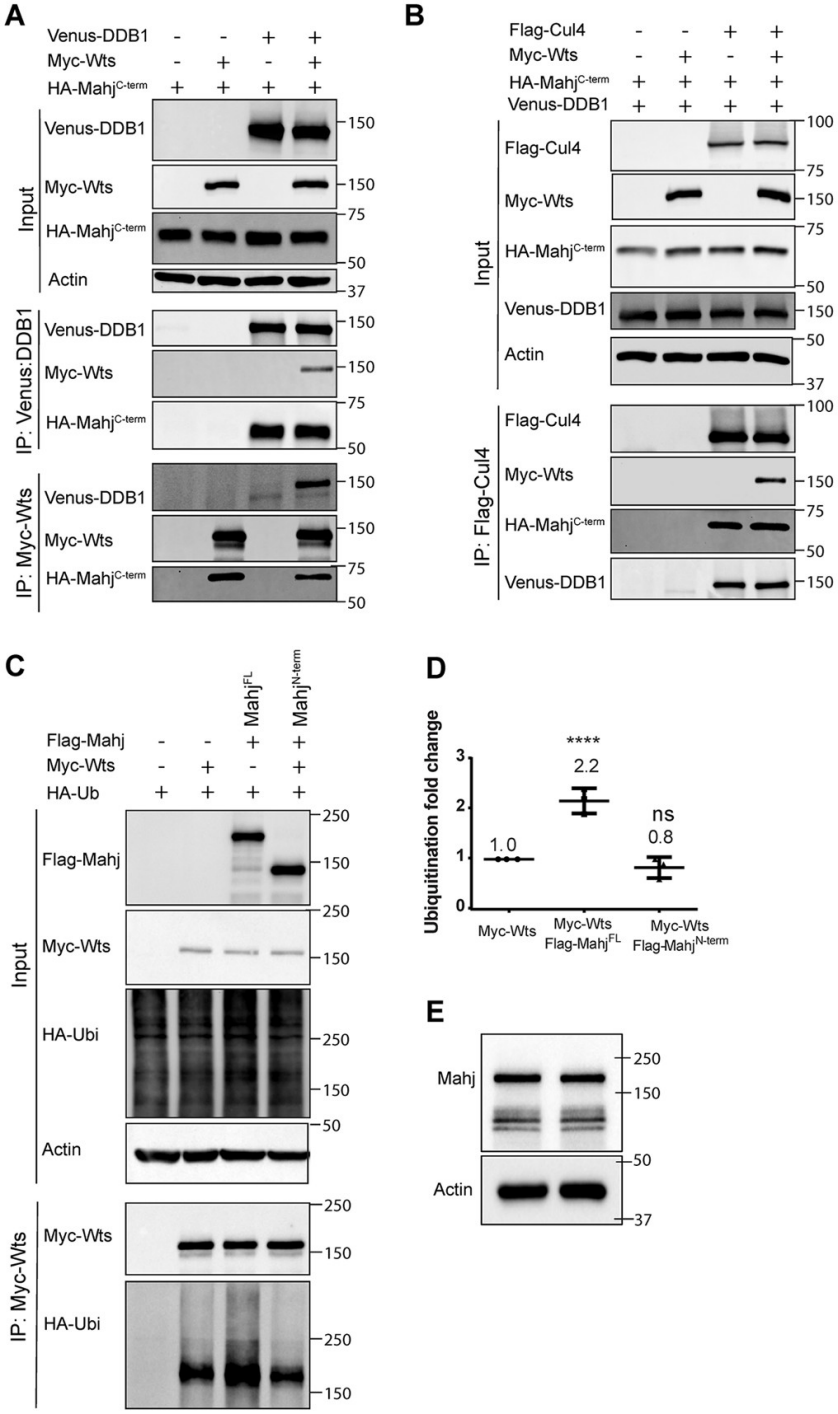
CRL4^Mahj^ forms a protein complex and promotes Wts ubiquitination. (A) Co-IP between Venus-DDB1 and Myc-Wts, in the presence of HA-Mahj^C-term^. (B) Co-IP between Flag-Cul4 and Myc-Wts, in the presence of HA-Mahj^C-term^ and Venus-DDB1. Anti-Myc, anti-GFP, or anti-Flag antibodies were used for IP, followed by western blotting probed with anti-Myc, anti-Flag-HRP, anti-HA, or anti-GFP antibodies. (C-D) In vivo ubiquitination assay in S2 cells. Heat-shock HA-Ubiquitin (“HA-Ub”), Myc-Wts, and Flag-Mahj^FL^ or Flag-Mahj^N-term^ were cotransfected into S2 cells. IP was conducted with anti-Myc antibody, and western blots were performed by using anti-HA and anti-Myc to detect ubiquitinated Wts and overall level of Myc-Wts, respectively. Actin served as a loading control. (D) Quantification for (C), *n* = 3. The ROD of the ubiquitinated Wts in the presence of Mahj co-overexpression was quantified on a densitometer and normalized to the ROD of ubiquitinated Wts in S2 cells without Mahj overexpression. (E) Protein extracts from untransfected S2 cells (in both lanes) were blotted with anti-Mahj antibody. Actin was used as a loading control. Data are presented as mean ± SD. **** for *P* ≤ 0.0001. The data underlying this figure can be found in S1 Data. CRL4, Cullin4-RING ligase; Cul4, Cullin4; DDB1, damaged DNA-binding protein 1; GFP, green fluorescent protein; HA, hemagglutinin; HRP, horseradish peroxidase; IP, immunoprecipitation; Mahj, Mahjong; ns, statistically nonsignificant; ROD, relative of density; Wts, Warts.

To investigate whether Wts is a potential substrate of CRL4^Mahj^ E3 ligase, we tested whether the adaptor DDB1 and the scaffold Cul4 of CRL4 could physically associate with Wts. We found that both Myc-Wts and HA-Mahj^C-term^ were detected in the immune complex following IP of Venus-DDB1 in S2 cells (Fig 5A). We did not detect a consistent association of Wts and DDB1 without ectopic expression of HA-Mahj^C-term^ (S5A Fig). Likewise, Flag-Cul4 was found in the same immune complex with Myc-Wts, Venus-DDB1, and HA-Mahj^C-term^ when HA-Mahj^C-term^ was expressed (Fig 5B). These observations suggest that Wts physically associates with both DDB1 and Cul4, likely through interactions with Mahj.

To further validate the physical association between Wts and different subunits of CRL4^Mahj^, we performed a proximity ligation assay (PLA) in situ. In the PLA, DNA-conjugated antibodies binding to two target proteins in close proximity can result in fluorescent foci produced as a result of specifically amplified DNA products [41]. The interaction between two proteins can be reliably assessed by quantifying the PLA fluorescent foci in cells coexpressing both Flag-tagged and Myc-tagged proteins (S5B Fig). In S2 cells coexpressing various control constructs, most cells (75.1%–97.2% of cells [*n* > 200], depending on the combinations of constructs) displayed no PLA foci, and the rest of the cells displayed weak PLA foci (1–10 foci); on average, there were 0–0.4 ± 0.2–0.8 PLA foci per cell (S5C–S5E Fig). By contrast, 97.9% of cells (*n* = 281) coexpressing Flag-Wts and Myc-Mahj showed PLA signals: 26.0%, 30.6%, and 41.3% of cell with strong (>20 foci), moderate (11–20 foci), and weak (1–10 foci) signals, respectively; they resulted in 16.2 ± 14.9 foci per cell (S5C–S5E Fig). Similarly, 97.8% of cells (*n* = 176) expressing Flag-Wts and Myc-DDB1 (*n* = 176) showed PLA signals: 10.1%, 27.0%, and 60.7% of the cells with strong, moderate, and weak PLA signals; and on average, these cells displayed 10.7 ± 9.94 PLA foci per cell (S5C–S5E Fig). The PLA signal analysis of cells coexpressing Flag-Wts and Myc-Cul4 (*n* = 157) also indicated positive physical interaction: 11.9 ± 10.5 PLA foci per cell and 97.5% cells showed strong, moderate, and weak PLA signals of 56.7%, 26.1%, and 14.6%, respectively (S5C–S5E Fig). These results provide a strong validation of evidence for physical interactions between Wts and various components of CRL4^Mahj^ from the co-IP experiments and mutant analysis. Taken together, these results indicate that CRL4^Mahj^ E3 ligase physically associates with Wts.

### Mahj promotes the ubiquitination of Wts

The association of Wts with CRL4^Mahj^ E3 ligase prompted us to test whether Mahj mediates the ubiquitination of Wts. To this end, we performed in vivo ubiquitination assays in S2 cells coexpressing HA-Ub, Myc-Wts, and Flag-Mahj^FL^ or Flag-Mahj^N-term^. Cells were treated with proteasome inhibitor MG132 to prevent Wts degradation by proteasomes. Following IP with anti-Myc antibody, Wts was ubiquitinated, and this was significantly enhanced by overexpression of full-length Mahj (Flag-Mahj^FL^) (Fig 5C and 5D). By contrast, Mahj^N-term^ lacking the HLH motif and WD40 domain failed to enhance the ubiquitination of Wts (Fig 5C and 5D). These results suggest that Wts can be ubiquitinated by CRL4^Mahj^. Ubiquitination of Wts without Flag-Mahj expression was also observed, presumably because of the endogenous Mahj expression in S2 cells (Fig 5E).

### CRL4^Mahj^ down-regulates Wts to promote NSC reactivation

**Fig 6:**
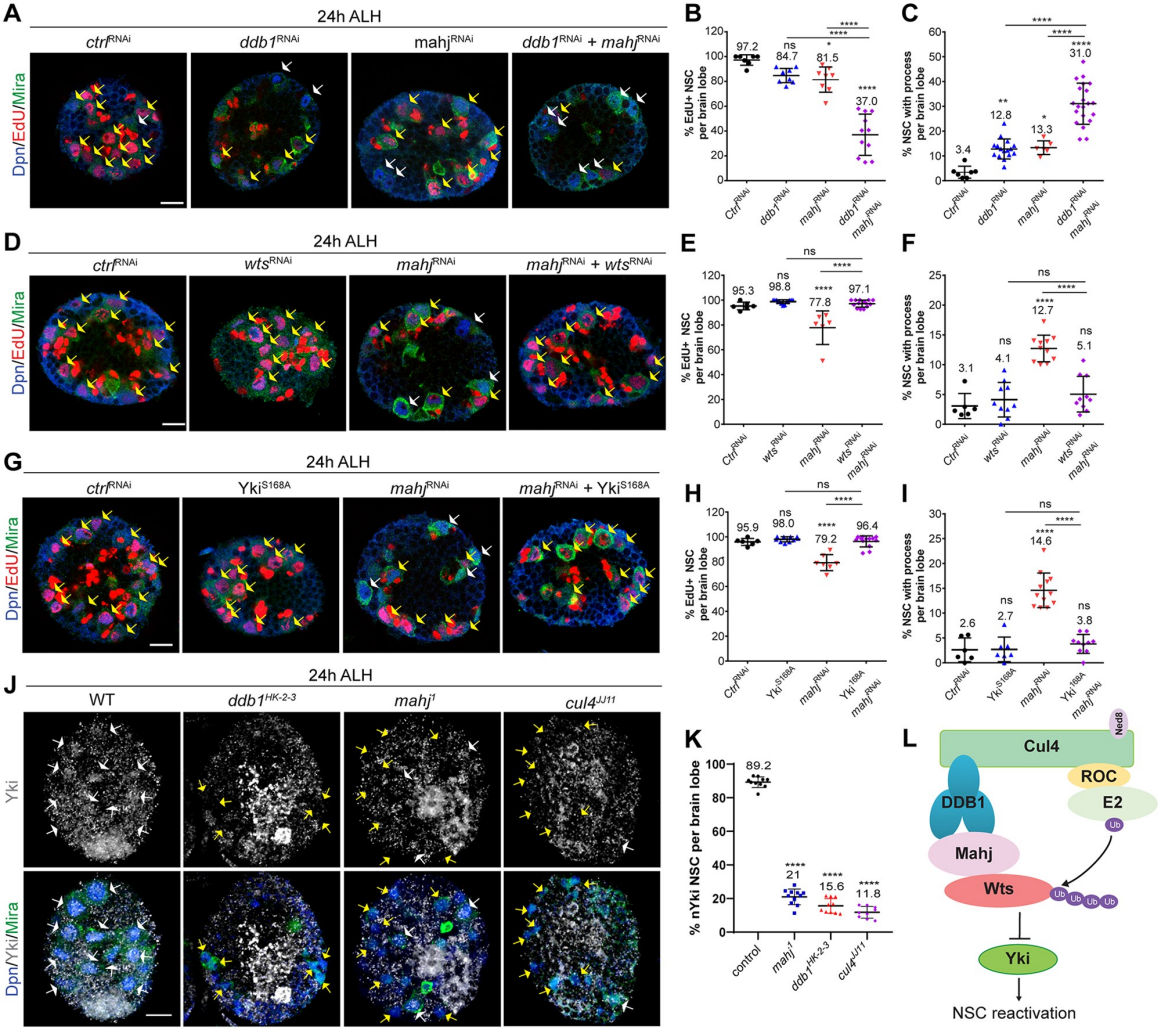
CRL4^Mahj^ inhibits Wts to promote NSC reactivation. (A) Larval NSCs in control (*ctrl*^RNAi^: *β*-*gal*^RNAi^), *ddb1*^RNAi^ control (VDRC#44974 +*β*-*gal*^RNAi^), *mahj*^RNAi^ control (BDSC#34912 + *β*-*gal*^RNAi^), and *mahj*^RNAi^ + *ddb1*^RNAi^ were labeled with Dpn, EdU, and Mira. (B-C) Quantification of Dpn^+^ Mira^+^ NSCs that are EdU^+^ or with cellular process. For (C), control: *n* = 7 brain lobes, t = 585 total NSCs; *ddb1*^RNAi^+*β*-*gal*^RNAi^: *n* = 17, t = 1,570; *mahj*^RNAi^ +*β*-*gal*^RNAi^: *n* = 6, t = 463; *mahj*^RNAi^ + *ddb1*^RNAi^: *n* = 21, t = 1,511. (D) Larval NSCs in *ctrl*^RNAi^, *wts*^RNAi^ (BDSC#34064+*β*-*gal*^RNAi^), *mahj*^RNAi^ +*β*-*gal*^RNAi^, and *mahj*^RNAi^ + *wts*^RNAi^ were labeled with Dpn, EdU, and Mira. (E-F) Quantification of Dpn^+^ Mira^+^ NSCs that are EdU^+^ or with cellular process. (G) Larval NSCs in *ctrl*^RNAi^, Yki^S168A^ + *β*-*gal*^RNAi^, *mahj*^RNAi^ +*β*-*gal*^RNAi^, and *mahj*^RNAi^ + Yki^S168A^ were labeled with Dpn, EdU, and Mira. (H-I) Quantification of the percentages of Dpn^+^ Mira^+^ NSCs that are EdU^+^ or with cellular process in (G). For (H), control: *n* = 6, t = 395; Yki^S168A^ + *β*-*gal*^RNAi^: *n* = 10, t = 596. For (I), control: *n* = 6, t = 489; Yki^S168A^ +*β*-*gal*^RNAi^: *n* = 7, t = 518. Yellow arrows, EdU^+^ NSCs. White arrows, EdU-negative NSCs. Driver is *insc*-Gal4, *tubulin*-Gal80^ts^. To balance the number of UAS elements across different genotypes, additional control UAS line (*β*-*gal*^RNAi^) was added to various RNAi lines in these experiments, resulting in a weaker phenotype compared with those without additional UAS control shown earlier in this study. (J) Larval NSCs in WT, *ddb1*^*HK-2-3*^, *mahj*^*1*^, and *cul4*^*JJ11*^ were labeled with Dpn, Mira, and Yki. White arrows, nuclear Yki-positive NSCs; yellow arrows, nuclear Yki-negative (or cytoplasmic Yki) NSCs. (K) Quantification of Dpn^+^ Mira^+^ NSCs that are nuclear Yki in various genotypes. (L) A working model illustrating the mechanism by which CRL4^Mahj^ promotes NSC reactivation. Data are presented as mean ± SD. **** for *P* ≤ 0.0001, *** for *P* ≤ 0.001, ** for *P* ≤ 0.01, * for *P* ≤ 0.05, and ns for P > 0.05. Scale bars: 10 μm. *n*, number of brain lobes; t, total number of NSCs. The data underlying this figure can be found in S1 Data. BDSC, Bloomington *Drosophila* Stock Center; CRL4, Cullin4-RING ligase; DDB1, damaged DNA-binding protein 1; Dpn; Deadpan; EdU, 5-ethynyl-2′-deoxyuridine; Mahj, Mahjong; Mira, Miranda; NSC, neural stem cell; RNAi, RNA interference; ROC, RING of Cullin; UAS, upstream activating sequence; Ub, ubiquitin; VDRC, Vienna Drosophila Resource Center; WT, wild type; Wts, Warts; Yki, Yorkie.

To seek further evidence that Mahj and DDB1 function together in regulating NSC reactivation, we examined whether *mahj* depletion could aggravate NSC reactivation defects observed upon *ddb1* depletion. Whereas 97.2% of control NSCs (*n* = 7, t = 374) were positive for EdU, 84.7% (*n* = 9, t = 575) of *ddb1*^RNAi^ and 81.5% (*n* = 8, t = 517) of *mah*j^RNAi^ controls incorporated EdU (Fig 6A and 6B). Upon simultaneous knockdown of *mahj* and *ddb1* by RNAi driven by *insc*-Gal4 driver, only 37.0% (*n* = 11, t = 499) of NSCs were EdU^+^ (Fig 6A and 6B), suggesting genetic enhancement between *mahj* and *ddb1*. Moreover, significantly more NSCs displaying a cellular process were observed in codepleted *mahj* and *ddb1* brains than in those of either *ddb1* or *mahj* knockdown (Fig 6A and 6C). These observations suggest that Mahj and DDB1 likely function in the same protein complex to promote NSC reactivation.

If CRL4^Mahj^ ubiquitinates and inhibits Wts to promote NSC reactivation, RNAi-mediated knockdown of *wts* should alleviate NSC reactivation defects induced by *mahj* depletion. Indeed, upon *wts* knockdown by the *insc*-Gal4 driver, 98.8% (*n* = 13, t = 817) of NSCs were positive for EdU, comparable to that of the control brains (95.3%, *n* = 6, t = 357) (Fig 6D and 6E). Whereas only 77.8% (*n* = 6, t = 402) of NSCs in *mahj* knockdown were EdU^+^ at 24 h ALH, upon simultaneous knockdown of both *mahj* and *wts* by *insc*-Gal4, 97.1% (*n* = 14, t = 1,008) of NSCs incorporated EdU (Fig 6D and 6E). Similarly, codepletion of *mahj* and *wts* resulted in fewer cellular process–retaining NSCs compared with that of *mahj* RNAi alone (Fig 6F; control: 3.1%, *n* = 6, t = 437; *wts*^RNAi^ control: 4.1%, *n* = 10, t = 786; *mahj*^RNAi^: 12.7%, *n* = 11, t = 959; *mahj*^RNAi^ + wts^RNAi^: 5.1%, *n* = 10, t = 739). These data suggest that *wts* knockdown significantly suppresses NSC reactivation defects observed in *mahj* RNAi brains. To maintain NSC quiescence, Wts phosphorylates and inactivates the transcriptional coactivator Yki to turn off expressions from Yki target genes [17,18]. Similar to *wts* knockdown, overexpression of a constitutively active form of Yki (Yki^S168A^) [42], which is insensitive to Wts-mediated phosphorylation and inactivation, significantly suppressed NSC reactivation defects observed in *mahj*^RNAi^ brains (Fig 6G–6I). Whereas 79.2% (*n* = 7, t = 518) EdU^+^ NSCs and 14.6% (*n* = 12, t = 965) NSCs with a cellular process were observed in *mahj*^RNAi^ control, upon Yki^S168A^ overexpression in *mahj* RNAi background, 96.4% (*n* = 13, t = 900) NSCs incorporated EdU^+^, and only 3.8% (*n* = 10, t = 634) of NSCs retained a cellular process (Fig 6G–6I). Furthermore, *wts* knockdown or overexpression of Yki^S168A^ could similarly alleviate the quiescent NSC phenotypes observed in *ddb1* or *cul4* knockdown (S6 Fig). Consistent with these observations, nuclear Yki localization in *ddb1*^*HK2-3*^ (15.6% with nuclear Yki, *n* = 10 brain lobes), *cul4*^*JJ11*^ (11.8%, *n* = 9), and *mahj*^*1*^ (21%, *n* = 11) was dramatically diminished in NSCs, compared with that in the WT control (Fig 6J and 6K; 89.2%, *n* = 10). Thus, these analyses support our conclusion that CRL4^Mahj^ promotes ubiquitination and down-regulation of Wts, thereby leading to the activation of Yki for NSC reactivation (Fig 6L).

## Discussion

Here, we demonstrate a novel role of an E3 ligase CRL4^Mahj^ consisting of two core subunits, namely DDB1 and Cul4, and a substrate-binding subunit Mahj during NSC reactivation. Whereas *mahj* loss of function results in a failure of NSCs reactivation, Mahj overexpression leads to premature reactivation of NSCs in the presence of dietary amino acids. Therefore, Mahj is both necessary and sufficient to promote NSC reactivation. Moreover, we show that CRL4^Mahj^ forms a protein complex with Wts, a core kinase of the evolutionarily conserved Hippo pathway that maintains NSC quiescence. Furthermore, Mahj promotes the ubiquitination of Wts. Lastly, loss of *wts* or overexpression of the constitutively active Yki largely rescues NSC reactivation defects resulting from depletion of various major components of CRL4^Mahj^. In summary, CRL4^Mahj^ complex targets its substrate Wts for ubiquitination and degradation to trigger NSC reactivation.

Although CRL4 E3s have diverse functions in DNA replication, cell cycle progression, and proliferation, CRL4’s function in *Drosophila* brain development was previously unknown. This study, for the first time (to our knowledge), elucidates the critical role of the *Drosophila* CRL4 E3 ligase in promoting NSC reactivation. Our finding is consistent with an earlier report that DDB1 deletion in the mouse brain leads to apoptosis of proliferating neural progenitor cells [43]. However, whether mouse DDB1 is required for proliferation/reactivation of these cells is unclear, as mouse DDB1 maintains viability and genomic integrity of dividing cells in the brain by its function in the DNA damage response [43]. In addition, human Cul4B is one of the most frequently mutated genes in X-linked mental retardation and cerebral malformations [31,32]. However, how loss of function of Cul4B causes these neurodevelopmental disorders is not well established. Knockdown of Cul4B in rat neural progenitor cells leads to cell cycle arrest at the G2/M phase [44], supporting our findings on the role of *Drosophila* Cul4 in promoting reactivation of NSCs. In this study, we demonstrated that *Drosophila* Cul4 in the complex with DDB1 is required for *Drosophila* NSC reactivation. We identified one underlying mechanism for Cul4 to promote NSC reactivation via inhibition of Hippo pathway. Future studies are warranted to determine if Cul4B also regulates behaviors of mammalian NSCs via a similar mechanism reported here.

Mahj, a substrate receptor of CRL4, was initially identified as a lethal giant larvae (Lgl)-binding protein that mediates cell competition in both *Drosophila* wing disc epithelium and mammalian cultured cells [36]. Our data indicate that Mahj plays a novel role in regulating NSC quiescence and reactivation: Mahj is both necessary and sufficient for promoting NSC reactivation. In contrast, neither DDB1 nor Cul4 overexpression resulted in premature NSC reactivation, suggesting that Mahj is a central component of CRL4 that regulates turnover of its substrates during NSC reactivation.

Hippo pathway plays a role in maintaining the quiescence of *Drosophila* NSCs [17,18]. Similarly, the role of the vertebrate Hippo pathway in regulating NSC proliferation has been established [45,46]. In the chick neural tube, overexpression of Yes-associated protein (YAP; an ortholog of *Drosophila* Yki) or inhibition of the upstream kinases of the Hippo pathway, such as LATS1/2 (orthologs of *Drosophila* Wts) and mammalian Ste20-like kinase (Mst)1/2 (orthologs of *Drosophila* Hippo) causes marked expansion of the NSC pool [46]. Mutations in genes encoding dachsous cadherin-related 1 (DCHS1) and FAT atypical cadherin 4 (FAT4), two activators of Hippo pathway, disrupt cerebral cortical development in human and mouse [45]. Therefore, the Hippo pathway plays an evolutionarily conserved role in maintaining quiescence and/or inhibiting proliferation of NSCs. Here, we show that down-regulation of the Hippo pathway core kinase Wts by the CRL4^Mahj^ E3 ligase complex triggers *Drosophila* NSC reactivation. Our results provide an *in vivo* regulatory mechanism of Hippo pathway during NSC quiescence and proliferation. In cancer cells, human CRL4^DCAF1^ also binds to LATS1/2 and inhibits the proteins in the nucleus [47]. Given the conservation of both the CRL4 E3 ligase and the Hippo pathway, the same regulation might exist in mammalian brains during the transition of NSC quiescence to proliferation. Our finding does not exclude the possibility that CRL4^Mahj^ can also act on additional substrates besides Wts to regulate NSC reactivation. The identification of the link between CRL4 and the Hippo pathway during the transition between NSC quiescence and reactivation could potentially open new strategies for the treatment of neurodevelopmental disorders by targeting the components of the Hippo pathway.

Recently, it was reported that the majority of quiescent *Drosophila* NSCs are arrested at G2 with a minority of quiescent NSCs at G0 phase [9], raising the importance of cell cycle progression during NSC reactivation. In addition to triggering NSC reactivation via cellular growth restriction [18], Hippo pathway might also regulate cell cycle to maintain NSC quiescence. The Hippo pathway has been implicated in cell cycle regulation. *Drosophila* Wts and mammalian LATS1 interact with cell division cycle 2 (Cdc2) and Cyclin A, suggesting a role in G2/M transition [48]. The oncogene Yki also regulates the cell division cycle, and Cyclin E is one of its targets during the development of *Drosophila* wing imaginal discs [49]. In addition, Cul4 regulates both G1 progression and G2/M transition in both *Drosophila* and human cells [44]. By contrast, *ddb1* RNAi in the eye imaginal discs induced an extra S phase in some cells that are normally postmitotic in the region more posterior to the morphogenetic furrow, which caused apoptosis of eye disc cells [50]. Therefore, DDB1 may have different functions in proliferation of imaginal discs and NSCs in the larval brain. Furthermore, DDB1 likely functions in both initial reactivation of NSCs and maintaining their proliferation. In contrast, *mahj* homozygous mutant larvae did not have any detectable morphological defects including the imaginal discs [36]. *Hs*DCAF1 regulates G2/M transition [51] and cell cycle progression [52]. Future study is warranted to pinpoint the function of CRL4 and the Hippo pathway in cell cycle regulation during NSC reactivation in both *Drosophila* and mammalian brains.

## Materials and methods

### Fly stocks and genetics

The following fly stocks were used in this study: UAS-DDB1, UAS-DDB1^RNAi-Res^, *ddb1*^HK-2-3^, *ddb1*^W197^ (generated in this study), *ddb1*^5-1^, *cul4*^*G1-3*^, *cul4*^*JJ11*^, UAS-Flag-Cul4, UAS-Flag-Cul4^KR^ (CT Chien); *mahj*^*1*^ [36]; UAS-Myc-Mahj, UAS-Myc-Mahj^R1120/1123E^ (this study). The following fly strains were obtained from BDSC: *β*-*gal*^RNAi^ (#50680), UAS-CD8-GFP (#32186), *wts*^RNAi^ (#34064), *mahj*^RNAi1^ (#34912) and UAS-Yki^S168A^ (#28818); RNAi lines including *ddb1*^RNAi^ (#44974) and *cul4*^RNAi1^ (#105668), *cul4*^RNAi2^ (#44829) and *mahj*^RNAi2^ (#110669) were obtained from the VDRC. Df(2R) XE2900 (#108418) is from the Kyoto Drosophila Genomics and Genetic Resource.

NSC drivers included *insc*-Gal4 (BDSC#8751; 1407-Gal4) or *insc*-Gal4, *tub*-Gal80^ts^. Glial driver was *repo*-Gal4 (BDSC# 7145). Ubiquitous driver was *tub*-Gal4 (BDSC#5138). UAS-Dcr2 (BDSC#24650) was used together with various RNAi stocks. *ddb1* and *mahj* RNAi knockdown efficiency was verified by immunostaining of anti-DDB1 and anti-Mahj antibodies in larval brains.

All experiments with mutants were carried out at 25 °C, and experiments for RNAi knockdown or overexpression were performed at 29 °C.

### Clonal analysis

MARCM clones were generated as previously described [33]. Briefly, larvae were heat shocked at 37 °C for 2 h shortly ALH and at 10–16 h after the first heat shock. Larvae were further aged for 3 d at 25 °C, and larval brains were dissected and processed for immunohistochemistry.

### EdU incorporation analysis

Larvae were fed with food containing 2% (0.2 mM) EdU from Click-iT EdU Imaging Kits (Invitrogen) for 4 h before dissection. The dissected larval brains were fixed with 4% EM-grade formaldehyde for 22 min, followed by three washes with 0.3% PBST, and blocked with 3% BSA in PBST for 30 min. Following blocking, detection of incorporated EdU by Alexa Fluor azide was according to the Click-iT EdU protocol (Invitrogen). The brains were then washed briefly twice and blocked with 3% BSA again for 20 min and subjected to immunohistochemistry.

### Generation of transgenic flies

UAS-DDB1, UAS-DDB1^RNAi-Res^, UAS-Myc-Mahj, UAS-Myc-Mahj^R1120/1123E^ transgenic flies were generated by P-element-mediated transformation (BestGene).

### PLA

PLA was performed on S2 cells that were transfected with the indicated plasmids using Effectene Transfection Reagent (QIAGEN). Cells (5 × 10^4^ per well) were seeded into 8-well chamber slides (Lab-Tek, Cat# 154941). The plasmids used were control Myc [53], control Flag [53], Flag-Wts, and Myc-Mahj, Myc-DDB1, and Myc-Cul4 with 50 ng of each plasmid per sample. Forty-eight hours after transfection, S2 cells were rinsed once with cold PBS, fixed with 4% formaldehyde in PBS for 15 min, and blocked in 5% BSA in PBST (0.1% Triton-X100) for 45 min. Cells were then incubated with primary antibodies (rabbit anti-Flag, 1:1,000; Sigma, Cat #7425; and mouse anti-Myc 1:1,000, Abcam Cat# ab32) at RT for 2 h before proceeding with Duolink PLA (Sigma-Aldrich) as described previously [53].

### Statistics

Statistical analysis was performed on GraphPad Prism 6. Unpaired two-tail *t* tests were used for comparison of two groups and one-way ANOVA for comparison of more than two samples. In ANOVA, Dunnett’s or Tukey’s post hoc test was used to obtain the *P* values for each pairwise comparison. A value of *P* < 0.05 was considered statistically significant. All the data are shown as mean ± SD. In this work, ns (statistically nonsignificant) indicates *P* > 0.05, * indicates *P* ≤ 0.05, ** indicates *P* ≤ 0.01, *** indicates *P* ≤ 0.001, and **** indicates *P* ≤ 0.0001. Comparisons are with WT control, unless otherwise indicated by a line between two genotypes. All experiments were performed with a minimum of two repeats in total.

## Supporting information

S1 Fig Related to Fig 1. DDB1 functions intrinsically during NSC reactivation. (A) MARCM clones of control (FRT82B), *ddb1*^*W197*^, *ddb1*^*HK-2-3*^ were labeled with CD8-GFP and costained with Dpn and Mira (upper panels) or Mira and EdU (lower panels). The percentage of EdU^+^ NSCs or NSCs with a process for the indicated genotypes are shown on the right. (B) Larval brains of WT and *ddb1*^*HK-2-3*^ at 96 h ALH stained with Mira and DDB1. Blue arrows, NSCs. (C, E) Larval brains in control (*β*-*gal*^RNA*i*^) and *ddb1*^RNAi^ (VDRC#44974) under *insc*-Gal4; UAS-Dcr2 driver were labeled with Dpn, EdU, and Mira (C) or Dpn and Mira (E). (D, F) Quantifications of various genotypes in (C, E). In (D), control: brain lobes *n* = 6, total NSCs t = 318; *ddb1*^RNAi1^, *n* = 16, t = 771. In (F), control: *n* = 4, t = 248; *ddb1*^RNAi1^: *n* = 6, t = 537. (G) Larval NSCs from control (homozygous *β*-*gal*^RNAi^), *ddb1* RNAi (VDRC#v44974) with *β*-*gal*^RNAi^, or *ddb1* RNAi overexpressing an RNAi-resistant *ddb1* transgene (*ddb1*^RNAi^ + DDB1^RNAi-res^) under *insc*-Gal4, *tub*-Gal80^ts^ were labeled with Dpn, EdU, and Mira. (H-I) Quantification Dpn^+^ Mira^+^ NSCs that are EdU^+^ (H) or with a cellular process (I). In (H), control: *n* = 10, t = 562; *ddb1*^RNAi^: *n* = 10, t = 586; *ddb1*^RNAi^ + DDB1^RNAi-res^: *n* = 13, t = 451. In (I), control: *n* = 12, t = 822; *ddb1*^RNAi^: *n* = 14, t = 815; *ddb1*^RNAi^ + DDB1^RNAi-res^: *n* = 9, t = 947. (J) Larval NSCs in control and *ddb1*^RNAi^ with UAS-Dcr2; *repo*-Gal4 were labeled with Dpn, EdU, and Mira. (K-L) Quantification of Dpn^+^ Mira^+^ NSCs that are EdU^+^ (K) or with a cellular process (L). In (K), control: *n* = 10, t = 425; *ddb1*^RNAi^: *n* = 11, t = 462. In (L), control: *n* = 5, t = 271; *ddb1*^RNAi^: *n* = 11, t = 695. Enlarged views of the white dotted boxes in the upper panels are shown in lower panels in (G) and (J). (M) Larval brains of *grh*-Gal4>UAS-CD8-GFP at 0 h ALH were labeled with DDB1 and an NSC marker Dpn. Blue arrows, Dpn^+^ NSCs. Data are presented as mean ± SD. ns for *P* > 0.05, *** for *P* ≤ 0.001, and **** for *P* ≤ 0.0001. Yellow arrows, proliferative NSCs; white arrows, quiescent NSCs. Arrowheads indicate the cellular process of quiescent NSCs. Scale bars, 10 μm. The data underlying this figure can be found in S1 Data. ALH, after larval hatching; Dcr2, Dicer 2; DDB1, damaged DNA-binding protein 1; Dpn, Deadpan; EdU, 5-ethynyl-2′-deoxyuridine; GFP, green fluorescent protein; MARCM, mosaic analysis of repressible cell marker; Mira, Miranda; ns, statistically nonsignificant; NSC, neural stem cell; RNAi, RNA interference; UAS, upstream activating sequence; VDRC, Vienna Drosophila Resource Center; WT, wild-type.
(TIF)

S2 Fig Related to Fig 2. Cul4 functions intrinsically during NSC reactivation. (A) Larval brains (96 h ALH) in control (*β*-*gal*^RNAi^), *cul4*^RNAi1^/VDRC#105668 with *repo*-Gal4; UAS-Dcr2 were stained with Dpn, Mira, and EdU. The lower panels are enlarged views of the white boxes in the upper panels. (B-C) Quantification of Dpn^+^ Mira^+^ NSCs that are EdU^+^ (B) or with cellular process (C). In (B), control: *n* = 10, t = 605; *cul4*^RNAi^, *n* = 17, t = 670. In (C), control, *n* = 5, t = 418; *cul4*^RNAi^, *n* = 10, t = 513. (D) Larval NSCs in control (*insc>β*-*gal*^RNAi^), *insc*>Cul4^WT^, and *insc*>Cul4^KR^ at 24 h ALH were labeled with Dpn, Mira, and EdU. (E-G) Quantification of Dpn^+^ Mira^+^ NSCs that are EdU^+^ (E), with cellular process (F) or PH3-positive (G). For (F), control, *n* = 7, t = 569; *insc*>Cul4^WT^: *n* = 6, t = 602; *insc*>Cul4^KR^, *n* = 11, t = 763. For (G), control, *n* = 7, t = 569; *insc*>Cul4^WT^, *n* = 6, t = 602; and *insc*>Cul4^KR^, *n* = 12, t = 847. (H) MB NSC lineages in larval brain on amino acid–depleted food from WT, *ddb1*^*HK-2-3*^, *ddb1*^*5-1*^, and *cul*^*G1-3*^ at 24 h ALH were labeled with EdU, Dpn (an NSC marker), and Dac (marks MB neurons surrounding MB NSCs). (I) Larval brains from control (*β*-*gal*^RNAi^), overexpression of InR^CA^, *ddb1*^*RNAi*^ control, and overexpression of InR^CA^ with *ddb1*^*RNAi*^ under *insc*-Gal4 on amino acid–depleted food at 24 h ALH were labeled with EdU. White dotted lines mark the brain lobe. (J) Quantification of EdU^+^ cells per brain lobe in various genotypes in (I). Data are presented as mean ± SD. **** for *P* ≤ 0.0001, *** for *P* ≤ 0.001, ** for *P* ≤ 0.01, * for *P* ≤ 0.05, and ns for *P* > 0.05. Yellow arrows, EdU^+^ NSCs. White arrows, NSCs without EdU or with process. Scale bars, 10 μm. The data underlying this figure can be found in S1 Data. ALH, after larval hatching; Cul4, Cullin 4; Dac, dachshund; Dcr2, Dicer 2; *ddb1*, damaged DNA-binding protein 1; Dpn, Deadpan; EdU, 5-ethynyl-2′-deoxyuridine; InR, Insulin receptor; MB, mushroom body; Mira, Miranda; ns, statistically nonsignificant; NSC, neural stem cell; PH3, phospho-Histone H3; RNAi, RNA interference; UAS, upstream activating sequence; VDRC, Vienna Drosophila Resource Center; WT, wild type.
(TIF)

S3 Fig Related to Fig 3. *mahj* loss of function in NSCs results in NSC reactivation defects. (A-F) At 24 h ALH, larval NSCs in control (*β-gal*^RNAi^), *mahj*^RNAi1^ (BDSC#34912), and *mahj*^RNAi2^ (VDRC#110669) under *insc*-Gal4; UAS-Dcr2 driver were labeled with Dpn and Mira in (A); Dpn, Mira, and EdU (C); or Dpn, Mira, and PH3 (E). Quantification of Dpn^+^ Mira^+^ NSCs that display cellular process or that are positive for EdU or for PH3 in (A, C, E) was shown in (B, D and F). Yellow arrows, EdU^+^ or PH3-positive NSCs. White arrows, EdU-negative or process-retaining NSCs. Arrowheads, the cellular process of quiescent NSCs. (G) At 24 h ALH, larval brains of WT and *mahj*^*1*^ stained with Mahj and NSC markers (Dpn and Mira). Blue arrows, NSCs. (H) Protein extracts from 24 h ALH whole-larvae lysate of WT and *mahj*^*1*^ were blotted with anti-Actin (loading control) and anti-Mahj antibodies. (I) Quantification of Mahj level in (H) was obtained by normalization of Mahj ROD to Actin ROD, *n* = 3. (J) At 48 h ALH, larval brains from WT, *mahj*^*1*^ mutants, and *mahj*^*1*^ mutants expressing either UAS-Myc-Mahj or UAS-Myc-Mahj^R1120/1123E^ driven by *insc*-Gal4 were labeled with Dpn, Mira, and EdU. Yellow arrows, EdU^+^ NSCs; white arrows, EdU-negative NSCs. (K) Quantification of Dpn^+^ Mira^+^ NSCs that are EdU^+^ in various genotypes in (J). (L) Larval NSCs in WT and *mahj*^*1*^*/Df* (Df [2R] XE-2900) transheterozygous mutants were labeled with EdU, Dpn, and Mira. (M-N) Quantification of NSCs that are EdU^+^ or with process of various genotypes in (L). (O) MB NSC lineages in larval brains from WT and *mahj*^*1*^ at 24 h ALH were labeled with EdU, Dpn, and Dac. The enlarged views of white dotted boxes are shown to the right. Yellow arrowheads, MB NSCs surrounded by Dac-positive MB neurons. (P) At 0 h ALH, larval brains of *grh*-gal4>UAS-CD8-GFP were labeled with Mahj and a NSC marker (Dpn). Blue arrows, NSCs. (Q) Quantification of number of NSCs in *ddb1*^−^, *cul4*^−^, and *mahj*^−^ mutant brains at 24 h ALH. Data are presented as mean ± SD. **** for *P* ≤ 0.0001, *** for *P* ≤ 0.001, ** for *P* ≤ 0.01, * for *P* ≤ 0.05, and ns for *P* > 0.05. Scale bars: 10 μm. The data underlying this figure can be found in S1 Data. ALH, after larval hatching; BDSC, Bloomington *Drosophila* Stock Center; *cul4*, Cullin 4; Dac, dachshund; Dcr2, Dicer 2; *ddb1*, damaged DNA-binding protein 1; Dpn, Deadpan; EdU, 5-ethynyl-2′-deoxyuridine; GFP, green fluorescent protein; Mahj, Mahjong; MB, mushroom body; Mira, Miranda; ns, statistically nonsignificant; NSC, neural stem cell; PH3, phospho-Histone H3; ROD, relative of density; UAS, upstream activating sequence; VDRC, Vienna Drosophila Resource Center; WT, wild type.
(TIF)

S4 Fig Related to Fig 4. Wts interacts with Mahj through its C terminus. (A-C) A homology model illustrating the interaction between *Dm*Mahj and *Dm*DDB1. (A) View of Mahj 1110–1441 aa (orange and yellow, ribbon depiction) and DDB1 (blue and green, ribbon depiction) heterodimers. (B) Detail of predicted interaction between residues R1120 and R1123 (red stick) side chains in the HLH motif of Mahj (yellow ribbon) and DDB1 (pale green ribbon). Relevant residues are depicted in stick models: R1220 and R1123 (Mahj, red), D1134 (Mahj, sand), and D788 (DDB1, blue). The predicted H-bonds are depicted by black dashed lines. Mahj R1120 and R1123 are corresponding to *Hs*DCAF1 R1053 and R1056, respectively. (C) Detail of predicted interaction between R1307 and R1343 (red stick) side chains in the WD40 domain of Mahj (yellow ribbon) and DDB1 (pale green ribbon). Relevant residues are depicted in stick models: R1307 and R1342 (Mahj, red), E1295 (Mahj, sand), and E201 (DDB1, blue). The predicted H-bonds are depicted by black dashed lines. Mahj R1307 and R1343 are corresponding to *Hs*DCAF1 R1247, R1283, respectively. (D) A schematic diagram illustrating different Wts domains and truncated Wts proteins. (E) Wts C-terminal fragment containing kinase domain interacts with Mahj. Co-IP between Myc-Mahj and Flag-Wts or indicated truncated constructs. Anti-Myc were used for IP, followed by western blotting probed with anti-Myc, anti-Flag, or anti-Actin antibodies. Actin served as a loading control. aa, amino acid; DDB1, damaged DNA-binding protein 1; *Dm*, *D*. *melanogaster*; HLH, helix-loop-helix; *Hs*DCAF1, *H*. *sapiens* DDB1-Cul4 associated factor 1; IP, immunoprecipitation; Mahj, Mahjong; Wts, Warts.
(TIF)

S5 Fig Related to Fig 5. Wts interacts with different components of CRL4^Mahj^ in S2 cells. (A) Co-IP between Flag-DDB1 and HA-Wts. S2 cells were cotransfected with Flag-DDB1 and HA-Wts or respective controls. Immunoprecipitation was performed using anti-Flag antibodies, and western blot was performed using anti-Flag or anti-HA antibodies. (B) A schematic illustration for PLA. (C) In situ PLA assay between Flag-Wts and either Myc-Mahj, Myc-DDB1, or Myc-Cul4. S2 cells transfected with the indicated plasmids were stained with Flag, Myc, and DAPI and detected for PLA signal (red). Cell outline was shown by DIC images. Scale bar, 10 μm. (D) Quantification graph showing the percentage of cells with PLA foci in (B). (E) Quantification for the average number of PLA foci per cell in (C). The number of cells used for quantification in (D, E) are coexpression of Flag-control + Myc-control (*n* = 272), Flag-Wts + Myc-control (*n* = 247), Flag-control + Myc-Mahj (*n* = 889), Flag-control + Myc-DDB1 (*n* = 284), Flag-control + Myc-Cul4 (*n* = 263), Flag-Wts + Myc-Mahj (*n* = 281), Flag-Wts + Myc-DDB1 (*n* = 176), and Flag-Wts + Myc-Cul4 (*n* = 157). The data underlying this figure can be found in S1 Data. CRL4, Cullin4-RING ligase; Cul4, Cullin 4; DDB1, damaged DNA-binding protein 1; DIC, differential interference contrast; HA, hemagglutinin; IP, immunoprecipitation; Mahj, Mahjong; PLA, proximity ligation assay; Wts, Warts.
(TIF)

S6 Fig Related to Fig 6. CRL4^Mahj^ inhibits Wts to promote NSC reactivation. (A) Larval NSCs in control (*ctrl*^RNAi^: *β-gal*^RNAi^), *wts*^RNAi^ +*β-gal*^RNAi^, *ddb1*^RNAi^ (VDRC#44974) +*β-gal*^RNAi^, and *ddb1*^RNAi^ + *wts*^RNAi^ were labeled with Dpn, Mira, and EdU. (B-C) Quantification of Dpn^+^ Mira^+^ NSCs that are EdU^+^ (B) or with cellular process (C). In (B), control (*β-gal*^RNAi^): brain lobes *n* = 8, total NSCs *t* = 517; *wts*^RNAi^+*β-gal*^RNAi^: *n* = 11, t = 705; *ddb1*^RNAi^+*β-gal*^RNAi^: *n* = 10, t = 647; *ddb1*^RNAi^ + *wts*^RNAi^: *n* = 15, t = 955. In (C), control (*β-gal*^RNAi^): *n* = 4, t = 320; *wts*^RNAi^+*β-gal*^RNAi^: *n* = 6, t = 485; *ddb1*^RNAi^+*β-gal*^RNAi^: *n* = 6, t = 598; *ddb1*^RNAi^ + *wts*^RNAi^: *n* = 16, t = 1,403. (D) Larval NSCs in *ctrl*^RNAi^ (*β-gal*^RNAi^), Yki^S168A^+*β-gal*^RNAi^, *ddb1*^RNAi^+ *β-gal*^RNAi^, and *ddb1*^RNAi^ + Yki^S168A^ were labeled with Dpn, Mira, and EdU. (E-F) Quantification of Dpn^+^ Mira^+^ NSCs that are EdU^+^ (E) or with cellular process (F). In (E), control (*β-gal*^RNAi^): *n* = 7, t = 388; Yki^S168A^+*β-gal*^RNAi^: *n* = 10, t = 645; *ddb1*^RNAi^+*β-gal*^RNAi^: *n* = 9, t = 551; *ddb1*^RNAi^ + Yki^S168A^: *n* = 11, t = 809. In (F), control (*β-gal*^RNAi^): *n* = 5, t = 429; Yki^S168A^+*β-gal*^RNAi^: *n* = 4, t = 255; *ddb1*^RNAi^+*β-gal*^RNAi^: *n* = 5, t = 357; *ddb1*^RNAi^ + Yki^S168A^: *n* = 9, t = 755. (G) Larval NSCs in *ctrl*^RNAi^ (*β-gal*^RNAi^), *wts*^RNAi^+*β-gal*^RNAi^, *cul4*^RNAi^ (VDRC#105668)+*β-gal*^RNAi^, and *cul4*^RNAi^ + *wts*^RNAi^ were labeled with Dpn and EdU. (H-I) Quantification of Dpn^+^ NSCs that are EdU^+^ or with Mira^+^ cellular process. In (H), control (*β-gal*^RNAi^): *n* = 13, t = 1,179; *wts*^RNAi^+*β-gal*^RNAi^: *n* = 19, t = 1,665; *cul4*^RNAi^+*β-gal*^RNAi^: *n* = 14, t = 1,073; *cul4*^RNAi^ + *wts*^RNAi^: *n* = 19, t = 1,404. In (I), control (*β-gal*^RNAi^): *n* = 8, t = 702; *wts*^RNAi^+*β-gal*^RNAi^: *n* = 8, t = 696; *cul4*^RNAi^+*β-gal*^RNAi^: *n* = 9, t = 772; *cul4*^RNAi^ + *wts*^RNAi^: *n* = 10, t = 847. (J) Larval NSCs in *ctrl*^RNAi^+*β-gal*^RNAi^, Yki^S168A^+*β-gal*^RNAi^, *cul4*^RNAi^+*β-gal*^RNAi^, and *cul4*^RNAi^ + Yki^S168A^ were labeled with Dpn and EdU. (K-L) Quantification of Dpn^+^ NSCs that are EdU^+^ or with Mira^+^ cellular process. In (K), control (*β-gal*^RNAi^): *n* = 12, t = 990; Yki^S168A^+*β-gal*^RNAi^: *n* = 11, t = 917; *cul4*^RNAi^+*β-gal*^RNAi^: *n* = 13, t = 1,056; *cul4*^RNAi^ + Yki^S168A^: *n* = 15, t = 950. In (L), control (*β-gal*^RNAi^): *n* = 7, t = 701; Yki^S168A^+*β-gal*^RNAi^: *n* = 4, t = 344; *cul4*^RNAi^+*β-gal*^RNAi^: *n* = 11, t = 974; *cul4*^RNAi^ + Yki^S168A^: *n* = 10, t = 895. Driver is *insc*-Gal4, *tub*-Gal80^ts^ (A-F), and *insc*-Gal4; UAS-Dcr2 (G-L). Note that to balance the number of UAS elements across different genotypes, additional control UAS line, *β-gal*^RNAi^ for (A-F) or CD8-GFP/BDSC#32186 for (G-L), was added to various RNAi lines, resulting in weaker phenotype in RNAi lines compared with those without additional UAS control shown earlier in this study. Yellow arrows, EdU^+^ NSCs. White arrows, EdU-negative NSCs. Data are presented as mean ± SD. **** for *P* ≤ 0.0001, *** for *P* ≤ 0.001, ** for *P* ≤ 0.01, * for *P* ≤ 0.05, and ns for *P* > 0.05. Scale bars, 10 μm. The data underlying this figure can be found in S1 Data. BDSC, Bloomington *Drosophila* Stock Center; CRL4, Cullin4-RING ligase; *ddb1*, damaged DNA-binding protein 1; Dpn, Deadpan; EdU, 5-ethynyl-2′-deoxyuridine; Mira, Miranda; ns, statistically nonsignificant; NSC, neural stem cell; RNAi, RNA interference; UAS, upstream activating sequence; VDRC, Vienna Drosophila Resource Center; Wts, Warts; Yki, Yorkie.
(TIF)

S1 Table List of primer for Gateway cloning. C-term, C-terminal fragment; F, forward; FL, full length; HLH+WD40, helix-loop-helix motif and WD40 domain; N-term, N-terminal fragment; R, reverse; sc, with stop codon; wo sc, without stop codon.
(XLSX)

S2 Table List of primers used for sequencing. F, forward; R, reverse.
(XLSX)

S3 Table List of primers used to generate RNAi-resistant UAS-DDB1 construct. DDB1, damaged DNA-binding protein 1; F, forward; R, reverse; RNAi, RNA interference; UAS, upstream activating sequence.
(XLSX)

S1 Text Supplementary materials and methods. (DOCX)

S1 Data Numerical data used in this study. (XLSX)

## Abbreviations

abo: abnormal oocyte
ALH: after larval hatching
Atg16: Autophagy-related 16
bchs: blue cheese
BDSC: Bloomington *Drosophila* Stock Center
Caf1-105: Chromatin assembly factor 1, p105 subunit
Caf1-55: Chromatin assembly factor 1, p55 subunit
Cdc2: cell division cycle 2
Co-IP: coimmunoprecipitation
CRL4: Cullin4-RING ligase
Cul4: Cullin4
Cul4B: Cullin4B
DCAF: DDB1-Cul4 associated factor
DCHS1: dachsous cadherin-related 1
DDA1: DET1- and DDB1-associated protein 1
DDB1: damaged DNA-binding protein 1
DILP: *Drosophila* insulin-like peptide
*Dm*: *Drosophila melanogaster*
Dpn: Deadpan
EdU: 5-ethynyl-2′-deoxyuridine
Elp2: Elongator complex protein 2
EMS: ethyl methane sulfonate
esc: extra sexcombs
FAT4: FAT atypical cadherin 4
GFP: green fluorescent protein
HLH: helix-loop-helix
*Hs*: *Homo sapiens*
InR: Insulin receptor
IP: immunoprecipitation
LATS: large tumor suppressor
Lgl: lethal giant larvae
Mahj: Mahjong
MARCM: mosaic analysis of repressible cell marker
MB: mushroom body
Mira: Miranda
Mst: mammalian Ste20-like kinase
NSC: neural stem cell
Nup43: Nucleoporin 43kD
Ohgt: Ohgata
Oseg6: Outer segment 6
PDB: Protein Data Bank
PH3: phospho-Histone H3
PI3K: phosphoinositide 3-kinase
PLA: proximity ligation assay
Poc1: Proteome of centrioles 1
RNAi: RNA interference
ROC: RING of Cullin
ROD: relative of density
slimb: supernumerary limbs
Trbl: Tribbles
U4-U6-60K: U4-U6 small nuclear riboprotein factor 60K
UAS: upstream activating sequence
VprBP: Vpr (HIV-1) Binding Protein
WDA: Will Decrease Acetylation
WMD: Wing Morphogenesis Defect
WT: wild type
Wts: Warts
YAP: Yes-associated protein
Yki: Yorkie
