# Supplementary figures and images for: CRL4Mahj E3 ubiquitin ligase promotes neural stem cell reactivation
Source: PLoS Biol. 2019 Jun 6;17(6):e3000276. doi: 10.1371/journal.pbio.3000276 (PMC6553684; doi:10.1371/journal.pbio.3000276)

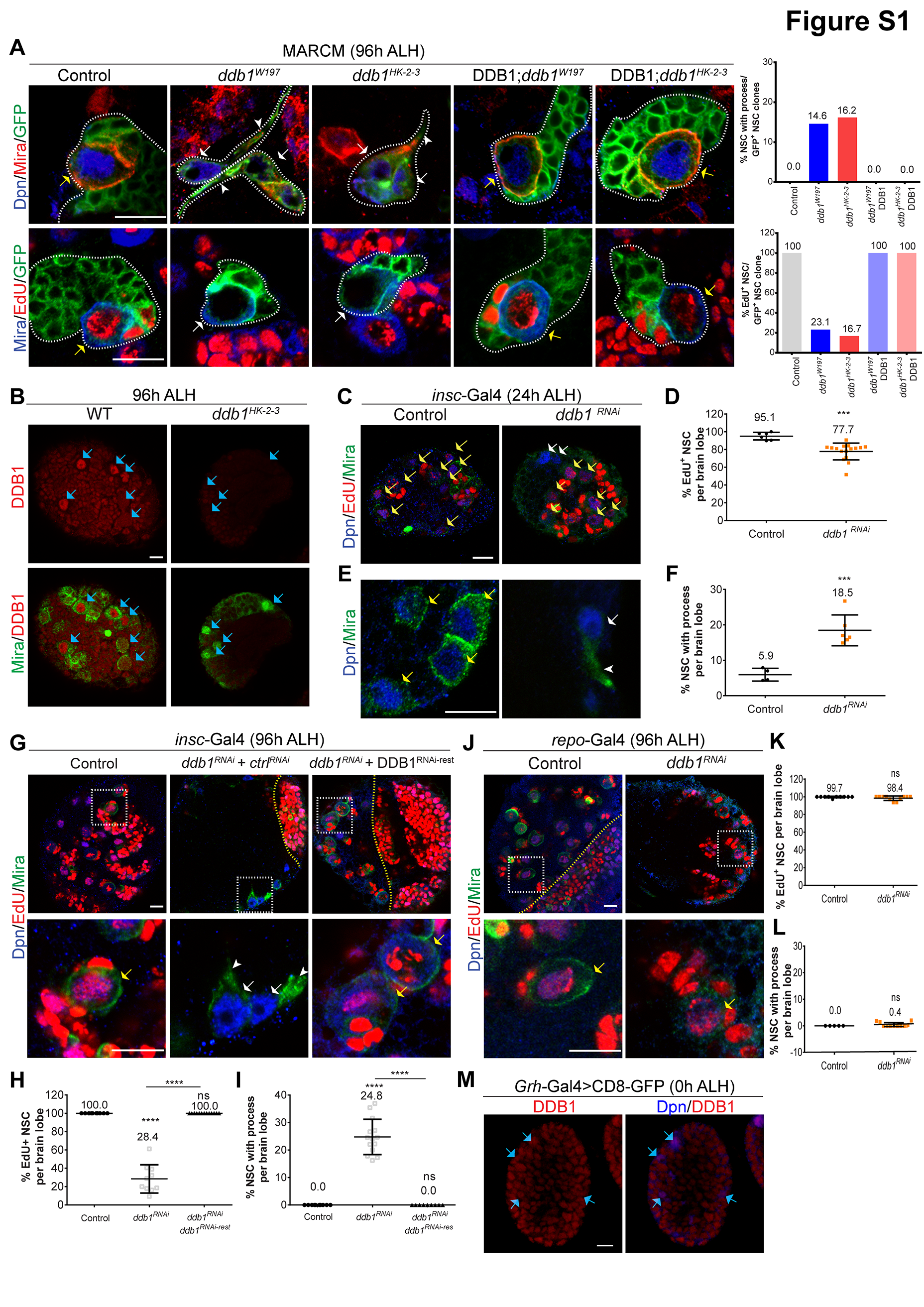

Supplement: S1 Fig — (A) MARCM clones of control (FRT82B), ddb1W197, ddb1HK-2-3 were labeled with CD8-GFP and costained with Dpn and Mira (upper panels) or Mira and EdU (lower panels). The percentage of EdU+ NSCs or NSCs with a process for the indicated genotypes are shown on the right. (B) Larval brains of WT and ddb1HK-2-3 at 96 h ALH stained with Mira and DDB1. Blue arrows, NSCs. (C, E) Larval brains in control (β-galRNAi) and ddb1RNAi (VDRC#44974) under insc-Gal4; UAS-Dcr2 driver were labeled with Dpn, EdU, and Mira (C) or Dpn and Mira (E). (D, F) Quantifications of various genotypes in (C, E). In (D), control: brain lobes n = 6, total NSCs t = 318; ddb1RNAi1, n = 16, t = 771. In (F), control: n = 4, t = 248; ddb1RNAi1: n = 6, t = 537. (G) Larval NSCs from control (homozygous β-galRNAi), ddb1 RNAi (VDRC#v44974) with β-galRNAi, or ddb1 RNAi overexpressing an RNAi-resistant ddb1 transgene (ddb1RNAi + DDB1RNAi-res) under insc-Gal4, tub-Gal80ts were labeled with Dpn, EdU, and Mira. (H-I) Quantification Dpn+ Mira+ NSCs that are EdU+ (H) or with a cellular process (I). In (H), control: n = 10, t = 562; ddb1RNAi: n = 10, t = 586; ddb1RNAi + DDB1RNAi-res: n = 13, t = 451. In (I), control: n = 12, t = 822; ddb1RNAi: n = 14, t = 815; ddb1RNAi + DDB1RNAi-res: n = 9, t = 947. (J) Larval NSCs in control and ddb1RNAi with UAS-Dcr2; repo-Gal4 were labeled with Dpn, EdU, and Mira. (K-L) Quantification of Dpn+ Mira+ NSCs that are EdU+ (K) or with a cellular process (L). In (K), control: n = 10, t = 425; ddb1RNAi: n = 11, t = 462. In (L), control: n = 5, t = 271; ddb1RNAi: n = 11, t = 695. Enlarged views of the white dotted boxes in the upper panels are shown in lower panels in (G) and (J). (M) Larval brains of grh-Gal4>UAS-CD8-GFP at 0 h ALH were labeled with DDB1 and an NSC marker Dpn. Blue arrows, Dpn+ NSCs. Data are presented as mean ± SD. ns for P > 0.05, *** for P ≤ 0.001, and **** for P ≤ 0.0001. Yellow arrows, proliferative NSCs; white arrows, quiescent NSCs. Arrowheads indicate the cellular [file pbio.3000276.s001.tif]

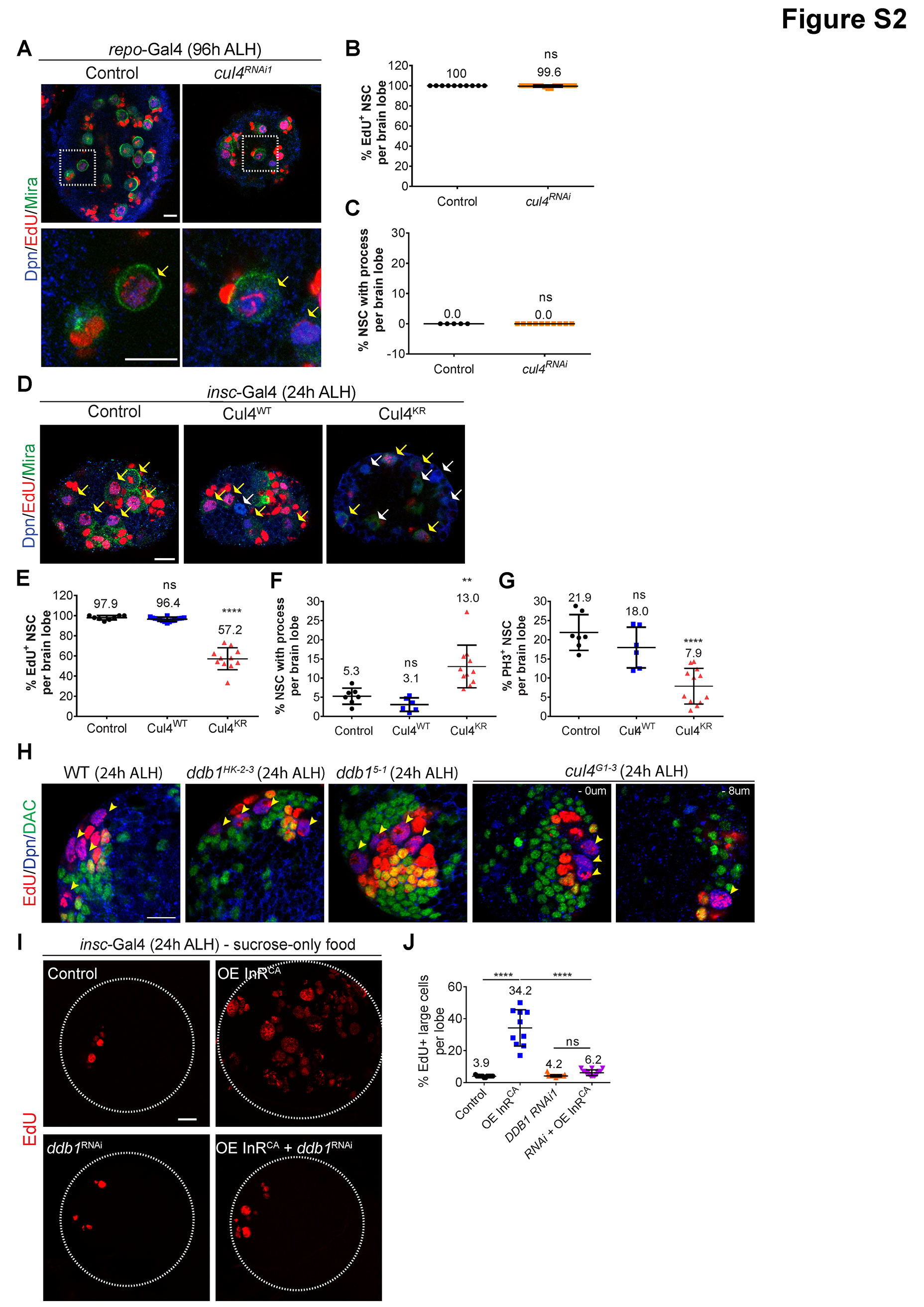

Supplement: S2 Fig — (A) Larval brains (96 h ALH) in control (β-galRNAi), cul4RNAi1/VDRC#105668 with repo-Gal4; UAS-Dcr2 were stained with Dpn, Mira, and EdU. The lower panels are enlarged views of the white boxes in the upper panels. (B-C) Quantification of Dpn+ Mira+ NSCs that are EdU+ (B) or with cellular process (C). In (B), control: n = 10, t = 605; cul4RNAi, n = 17, t = 670. In (C), control, n = 5, t = 418; cul4RNAi, n = 10, t = 513. (D) Larval NSCs in control (insc>β-galRNAi), insc>Cul4WT, and insc>Cul4KR at 24 h ALH were labeled with Dpn, Mira, and EdU. (E-G) Quantification of Dpn+ Mira+ NSCs that are EdU+ (E), with cellular process (F) or PH3-positive (G). For (F), control, n = 7, t = 569; insc>Cul4WT: n = 6, t = 602; insc>Cul4KR, n = 11, t = 763. For (G), control, n = 7, t = 569; insc>Cul4WT, n = 6, t = 602; and insc>Cul4KR, n = 12, t = 847. (H) MB NSC lineages in larval brain on amino acid–depleted food from WT, ddb1HK-2-3, ddb15-1, and culG1-3 at 24 h ALH were labeled with EdU, Dpn (an NSC marker), and Dac (marks MB neurons surrounding MB NSCs). (I) Larval brains from control (β-galRNAi), overexpression of InRCA, ddb1RNAi control, and overexpression of InRCA with ddb1RNAi under insc-Gal4 on amino acid–depleted food at 24 h ALH were labeled with EdU. White dotted lines mark the brain lobe. (J) Quantification of EdU+ cells per brain lobe in various genotypes in (I). Data are presented as mean ± SD. **** for P ≤ 0.0001, *** for P ≤ 0.001, ** for P ≤ 0.01, * for P ≤ 0.05, and ns for P > 0.05. Yellow arrows, EdU+ NSCs. White arrows, NSCs without EdU or with process. Scale bars, 10 μm. The data underlying this figure can be found in S1 Data. ALH, after larval hatching; Cul4, Cullin 4; Dac, dachshund; Dcr2, Dicer 2; ddb1, damaged DNA-binding protein 1; Dpn, Deadpan; EdU, 5-ethynyl-2′-deoxyuridine; InR, Insulin receptor; MB, mushroom body; Mira, Miranda; ns, statistically nonsignificant; NSC, neural stem cell; PH3, phospho-Histone H3; RNAi, RNA interference; UAS, upstream activating [file pbio.3000276.s002.tif]

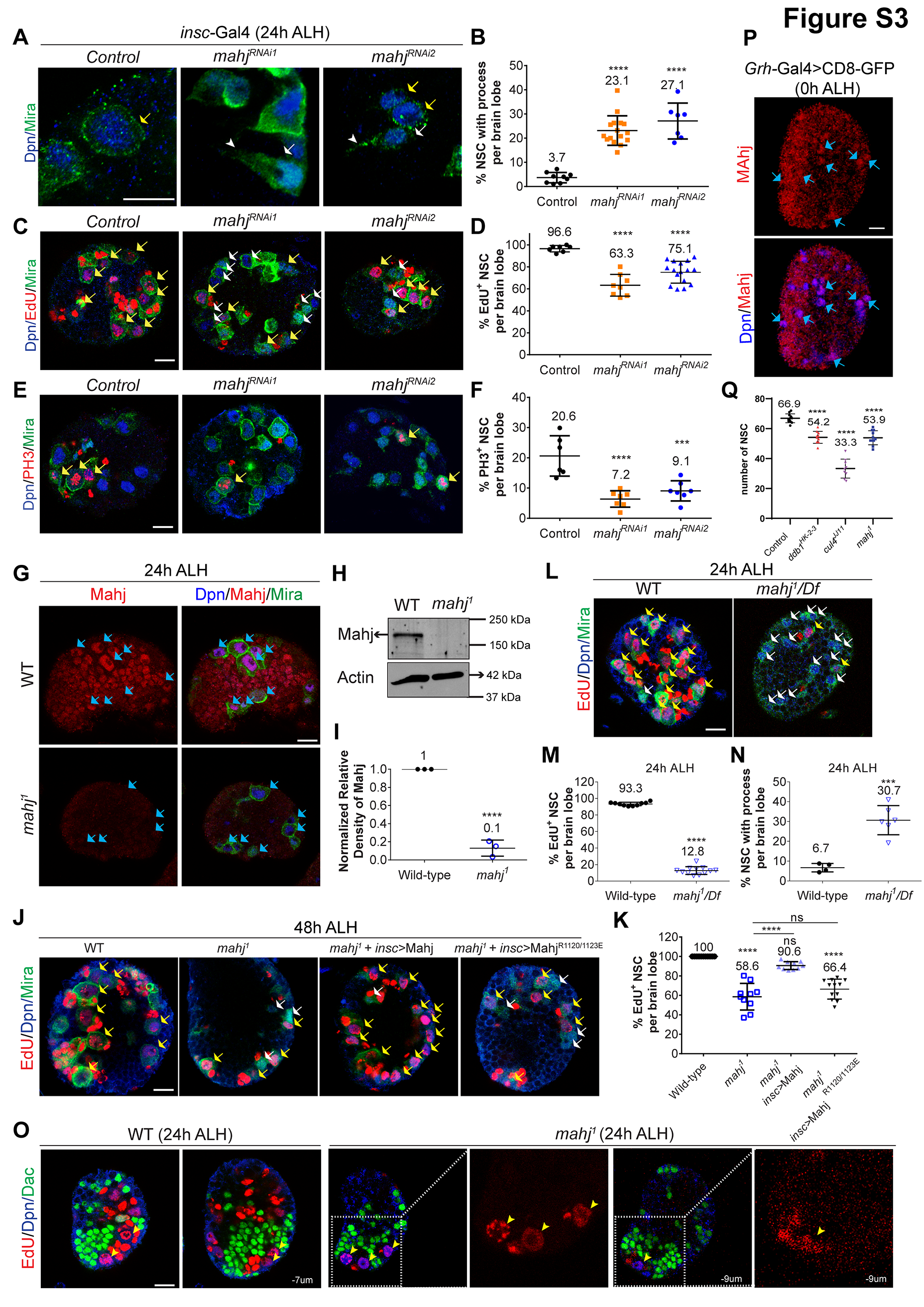

Supplement: S3 Fig — (A-F) At 24 h ALH, larval NSCs in control (β-galRNAi), mahjRNAi1 (BDSC#34912), and mahjRNAi2 (VDRC#110669) under insc-Gal4; UAS-Dcr2 driver were labeled with Dpn and Mira in (A); Dpn, Mira, and EdU (C); or Dpn, Mira, and PH3 (E). Quantification of Dpn+ Mira+ NSCs that display cellular process or that are positive for EdU or for PH3 in (A, C, E) was shown in (B, D and F). Yellow arrows, EdU+ or PH3-positive NSCs. White arrows, EdU-negative or process-retaining NSCs. Arrowheads, the cellular process of quiescent NSCs. (G) At 24 h ALH, larval brains of WT and mahj1 stained with Mahj and NSC markers (Dpn and Mira). Blue arrows, NSCs. (H) Protein extracts from 24 h ALH whole-larvae lysate of WT and mahj1 were blotted with anti-Actin (loading control) and anti-Mahj antibodies. (I) Quantification of Mahj level in (H) was obtained by normalization of Mahj ROD to Actin ROD, n = 3. (J) At 48 h ALH, larval brains from WT, mahj1 mutants, and mahj1 mutants expressing either UAS-Myc-Mahj or UAS-Myc-MahjR1120/1123E driven by insc-Gal4 were labeled with Dpn, Mira, and EdU. Yellow arrows, EdU+ NSCs; white arrows, EdU-negative NSCs. (K) Quantification of Dpn+ Mira+ NSCs that are EdU+ in various genotypes in (J). (L) Larval NSCs in WT and mahj1/Df (Df [2R] XE-2900) transheterozygous mutants were labeled with EdU, Dpn, and Mira. (M-N) Quantification of NSCs that are EdU+ or with process of various genotypes in (L). (O) MB NSC lineages in larval brains from WT and mahj1 at 24 h ALH were labeled with EdU, Dpn, and Dac. The enlarged views of white dotted boxes are shown to the right. Yellow arrowheads, MB NSCs surrounded by Dac-positive MB neurons. (P) At 0 h ALH, larval brains of grh-gal4>UAS-CD8-GFP were labeled with Mahj and a NSC marker (Dpn). Blue arrows, NSCs. (Q) Quantification of number of NSCs in ddb1−, cul4−, and mahj− mutant brains at 24 h ALH. Data are presented as mean ± SD. **** for P ≤ 0.0001, *** for P ≤ 0.001, ** for P ≤ 0.01, * for P ≤ 0.05, and ns for P > 0.05. Scale ba [file pbio.3000276.s003.tif]

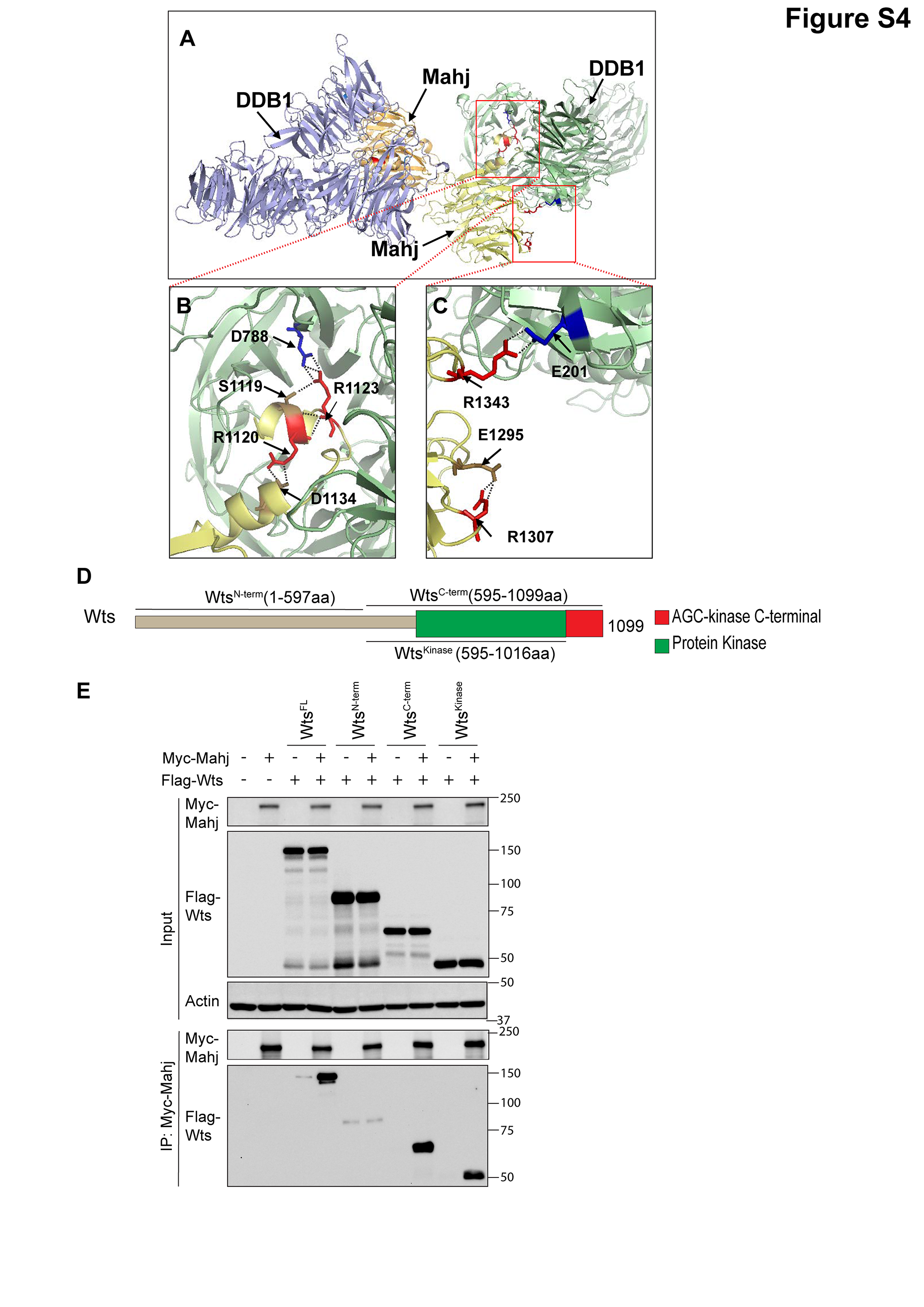

Supplement: S4 Fig — (A-C) A homology model illustrating the interaction between DmMahj and DmDDB1. (A) View of Mahj 1110–1441 aa (orange and yellow, ribbon depiction) and DDB1 (blue and green, ribbon depiction) heterodimers. (B) Detail of predicted interaction between residues R1120 and R1123 (red stick) side chains in the HLH motif of Mahj (yellow ribbon) and DDB1 (pale green ribbon). Relevant residues are depicted in stick models: R1220 and R1123 (Mahj, red), D1134 (Mahj, sand), and D788 (DDB1, blue). The predicted H-bonds are depicted by black dashed lines. Mahj R1120 and R1123 are corresponding to HsDCAF1 R1053 and R1056, respectively. (C) Detail of predicted interaction between R1307 and R1343 (red stick) side chains in the WD40 domain of Mahj (yellow ribbon) and DDB1 (pale green ribbon). Relevant residues are depicted in stick models: R1307 and R1342 (Mahj, red), E1295 (Mahj, sand), and E201 (DDB1, blue). The predicted H-bonds are depicted by black dashed lines. Mahj R1307 and R1343 are corresponding to HsDCAF1 R1247, R1283, respectively. (D) A schematic diagram illustrating different Wts domains and truncated Wts proteins. (E) Wts C-terminal fragment containing kinase domain interacts with Mahj. Co-IP between Myc-Mahj and Flag-Wts or indicated truncated constructs. Anti-Myc were used for IP, followed by western blotting probed with anti-Myc, anti-Flag, or anti-Actin antibodies. Actin served as a loading control. aa, amino acid; DDB1, damaged DNA-binding protein 1; Dm, D. melanogaster; HLH, helix-loop-helix; HsDCAF1, H. sapiens DDB1-Cul4 associated factor 1; IP, immunoprecipitation; Mahj, Mahjong; Wts, Warts. (TIF) [file pbio.3000276.s004.tif]

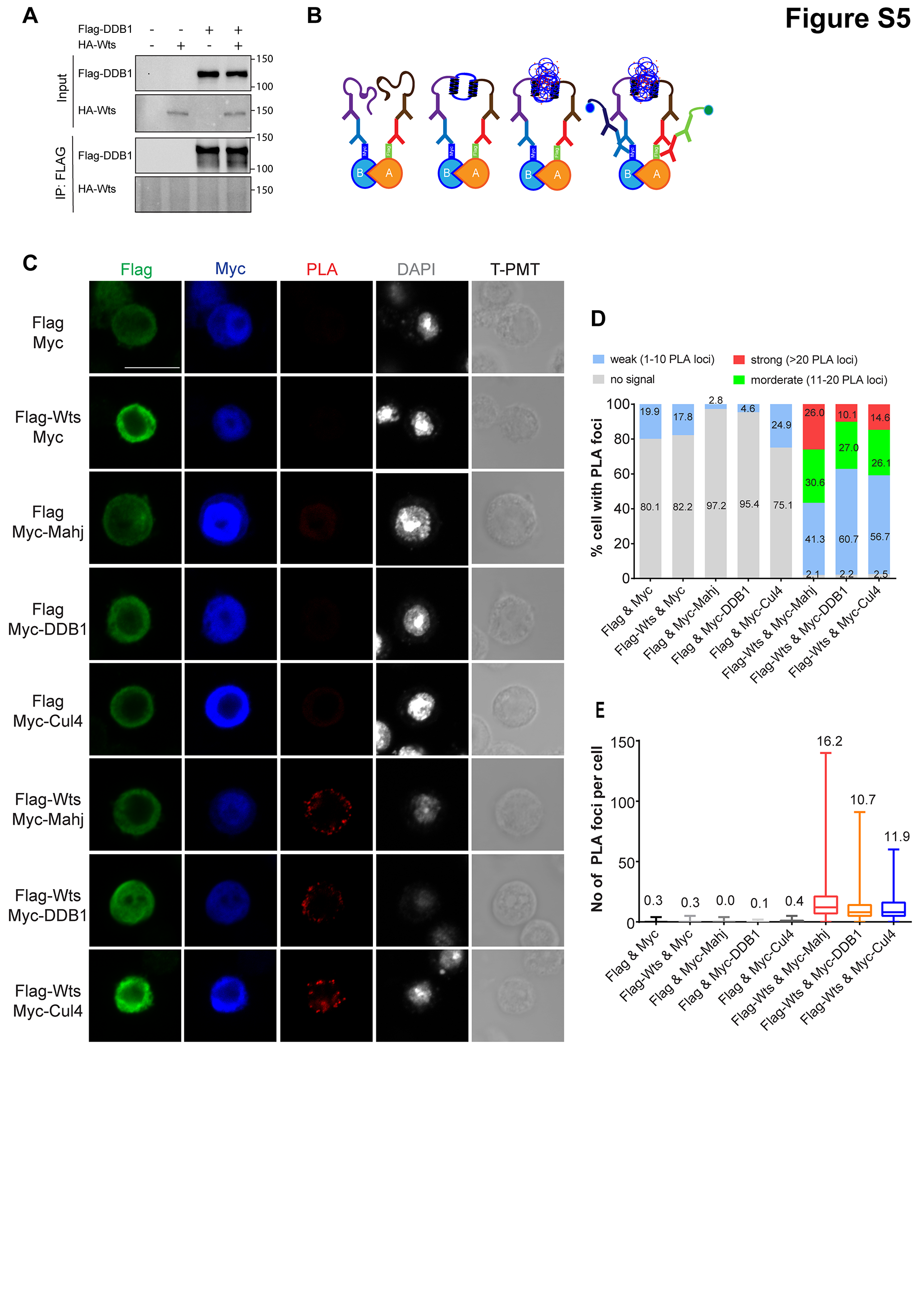

Supplement: S5 Fig — (A) Co-IP between Flag-DDB1 and HA-Wts. S2 cells were cotransfected with Flag-DDB1 and HA-Wts or respective controls. Immunoprecipitation was performed using anti-Flag antibodies, and western blot was performed using anti-Flag or anti-HA antibodies. (B) A schematic illustration for PLA. (C) In situ PLA assay between Flag-Wts and either Myc-Mahj, Myc-DDB1, or Myc-Cul4. S2 cells transfected with the indicated plasmids were stained with Flag, Myc, and DAPI and detected for PLA signal (red). Cell outline was shown by DIC images. Scale bar, 10 μm. (D) Quantification graph showing the percentage of cells with PLA foci in (B). (E) Quantification for the average number of PLA foci per cell in (C). The number of cells used for quantification in (D, E) are coexpression of Flag-control + Myc-control (n = 272), Flag-Wts + Myc-control (n = 247), Flag-control + Myc-Mahj (n = 889), Flag-control + Myc-DDB1 (n = 284), Flag-control + Myc-Cul4 (n = 263), Flag-Wts + Myc-Mahj (n = 281), Flag-Wts + Myc-DDB1 (n = 176), and Flag-Wts + Myc-Cul4 (n = 157). The data underlying this figure can be found in S1 Data. CRL4, Cullin4-RING ligase; Cul4, Cullin 4; DDB1, damaged DNA-binding protein 1; DIC, differential interference contrast; HA, hemagglutinin; IP, immunoprecipitation; Mahj, Mahjong; PLA, proximity ligation assay; Wts, Warts. (TIF) [file pbio.3000276.s005.tif]

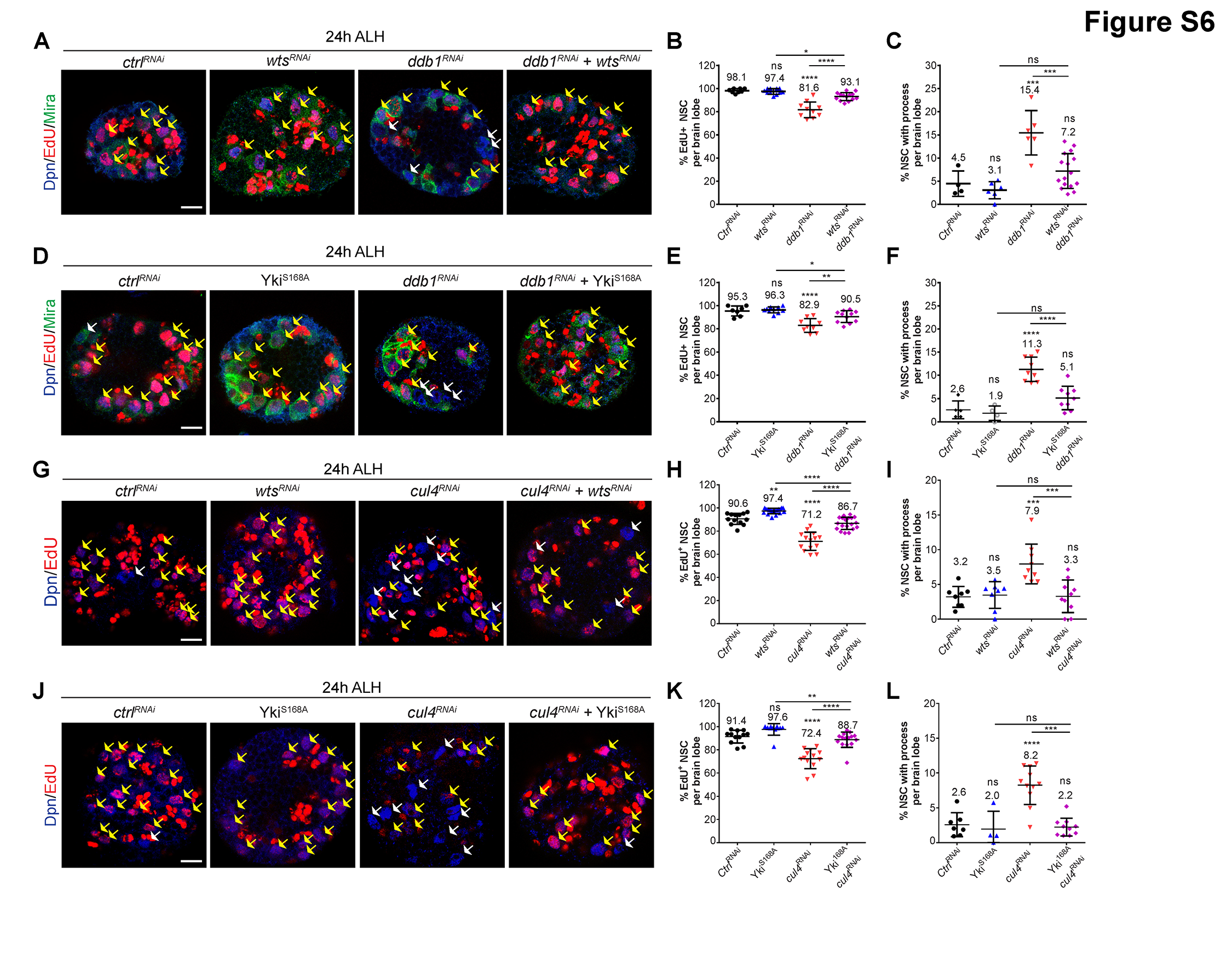

Supplement: S6 Fig — (A) Larval NSCs in control (ctrlRNAi: β-galRNAi), wtsRNAi +β-galRNAi, ddb1RNAi (VDRC#44974) +β-galRNAi, and ddb1RNAi + wtsRNAi were labeled with Dpn, Mira, and EdU. (B-C) Quantification of Dpn+ Mira+ NSCs that are EdU+ (B) or with cellular process (C). In (B), control (β-galRNAi): brain lobes n = 8, total NSCs t = 517; wtsRNAi+β-galRNAi: n = 11, t = 705; ddb1RNAi+β-galRNAi: n = 10, t = 647; ddb1RNAi + wtsRNAi: n = 15, t = 955. In (C), control (β-galRNAi): n = 4, t = 320; wtsRNAi+β-galRNAi: n = 6, t = 485; ddb1RNAi+β-galRNAi: n = 6, t = 598; ddb1RNAi + wtsRNAi: n = 16, t = 1,403. (D) Larval NSCs in ctrlRNAi (β-galRNAi), YkiS168A+β-galRNAi, ddb1RNAi+ β-galRNAi, and ddb1RNAi + YkiS168A were labeled with Dpn, Mira, and EdU. (E-F) Quantification of Dpn+ Mira+ NSCs that are EdU+ (E) or with cellular process (F). In (E), control (β-galRNAi): n = 7, t = 388; YkiS168A+β-galRNAi: n = 10, t = 645; ddb1RNAi+β-galRNAi: n = 9, t = 551; ddb1RNAi + YkiS168A: n = 11, t = 809. In (F), control (β-galRNAi): n = 5, t = 429; YkiS168A+β-galRNAi: n = 4, t = 255; ddb1RNAi+β-galRNAi: n = 5, t = 357; ddb1RNAi + YkiS168A: n = 9, t = 755. (G) Larval NSCs in ctrlRNAi (β-galRNAi), wtsRNAi+β-galRNAi, cul4RNAi (VDRC#105668)+β-galRNAi, and cul4RNAi + wtsRNAi were labeled with Dpn and EdU. (H-I) Quantification of Dpn+ NSCs that are EdU+ or with Mira+ cellular process. In (H), control (β-galRNAi): n = 13, t = 1,179; wtsRNAi+β-galRNAi: n = 19, t = 1,665; cul4RNAi+β-galRNAi: n = 14, t = 1,073; cul4RNAi + wtsRNAi: n = 19, t = 1,404. In (I), control (β-galRNAi): n = 8, t = 702; wtsRNAi+β-galRNAi: n = 8, t = 696; cul4RNAi+β-galRNAi: n = 9, t = 772; cul4RNAi + wtsRNAi: n = 10, t = 847. (J) Larval NSCs in ctrlRNAi+β-galRNAi, YkiS168A+β-galRNAi, cul4RNAi+β-galRNAi, and cul4RNAi + YkiS168A were labeled with Dpn and EdU. (K-L) Quantification of Dpn+ NSCs that are EdU+ or with Mira+ cellular process. In (K), control (β-galRNAi): n = 12, t = 990; YkiS168A+β-galRNAi: n = 11, t = 917; cul4RNAi+β-galRNAi: n = 13, t [file pbio.3000276.s006.tif]
