# Supplementary material for: CRL4Mahj E3 ubiquitin ligase promotes neural stem cell reactivation
Source: PLoS Biol. 2019 Jun 6;17(6):e3000276. doi: 10.1371/journal.pbio.3000276 (PMC6553684; doi:10.1371/journal.pbio.3000276)
Supplement: S1 Text — (DOCX) [file pbio.3000276.s010.docx]

**Supplementary Materials and Methods**

**Genetic screen on EMS mutants**

Genetic lesions were induced by 25 mM ethyl methane sulfonate (EMS) on *w^1118^*, FRT82B males. Lethal mutations on Chromosome III were balanced using a *TM6B,* *Tb, Hu* balancer. Totally 1194 lethal stocks were established and crossed to the MARCM driver *hs-Flp*, *elav-Gal4*; *UAS-LacZ*, *UAS-CD8::GFP/CyO; FRT82B, tubulin-Gal80/TM6B, Tb, Hu* to generate MARCM NSC clones. From this screen, *ddb1^HK-2-3^* and *ddb1^W197^* mutants were isolated for their phenotypes in the MARCM clones in the larval brains.

**Immunochemistry**

Dissected larval brains were fixed with 4% EM-grade formaldehyde in PBS for 22 minutes (min) at room-temperature (RT) followed by three washes in PBST (0.3% (PBS supplemented with 0.3% Triton-X). Fixed brains were blocked with 3% BSA in 0.3% PBST and incubated with primary antibody in blocking buffer overnight at 4°C. After three washes in 0.3% PBST, larval brains were incubated with secondary antibody in 0.3% PBST for 90 min, followed by two washes with 0.3% PBST, and mounted in a glycerol based mounting medium (10 mg/ml of p-Phenylenediamine in PBS, 1:10 dilution with glycerol). For staining of DNA, ToPro-3 in 0.3% PBST was added to specimens for 30 min just before adding mounting medium. Samples were imaged on a Zeiss LSM 710 confocal microscope and images were processed with Zeiss software.

Primary antibodies used were: guinea-pig anti-Dpn (1:1000; J. Skeath), mouse anti-Mira (1:50, F. Matsuzaki), rabbit anti-GFP (1:500, Molecular Probes, Cat #A21311), rabbit anti-PH3 (1:200, Sigma, Cat #06-570), mouse anti-Dac (1:5, DSHB, Cat #mAbdac2-3), rabbit anti-GFP (1:2000 with 50% glycerol, F. Yu), rabbit anti-DDB1 (1:500 with 50% glycerol, this study), guinea pig anti-Mahj (1:500 with 50% glycerol, this study) and rabbit anti-Yki (1:50, L. Zhang). DNA was labelled by ToPro-3 (1:5000, Invitrogen, Cat #T3605).

**Molecular cloning**

Expressed-sequence tags (EST) obtained from the *Drosophila* Genomics Resource Center (DGRC) were: LD08715 (*ddb1*), LP02965 (*cul4*), RE10692 (*mahj*). For RE10692, there was a mutation (GGGG) at exon 4 compared to (CTGGA) for the sequence of Mahj-PA obtained from Flybase. To correct this mutation, PCR-based mutagenesis was performed on RE10692. Briefly, a forward primer (Mahj cor RE10692 F1) and a reverse primer (Mahj cor RE10692 R1) containing the correct sequence (CTGGA) were used together with the reverse (Mahj BP sc R1) and the forward (Mahj BP F) primers, respectively, to amply N-terminal and C-terminal fragments of mahj CDS. These two fragments contained an overlap of ~25 nucleotides (including the correct sequence) and were used as templates for two-template PCR to amplify a full-length *mahj* CDS for BP cloning (see below). pA-HA-Wts (a gift from DJ Pan) was used to obtain a full-length coding sequence of Wts. Full length or truncated fragments of DDB1, Cul4, Mahj, Wts were amplified by PCR and cloned into the pENTR vector using the pENTR^TM^ Directional TOPO® Cloning Kit (Invitrogen, Cat# K2400-20) or pDONR221 by the BP Recombination Reaction (Invitrogen, Cat# 12535-019). All the gene information for this study was obtained from Flybase ([www.flybase.org](http://www.flybase.org)). Entry clones were subsequently cloned into various destination vectors using LR clonase II (Invitrogen) according to the manufacturer’s protocol. All primers (S1-S3 Tables) were synthesized by Integrated DNA Technology (IDT), Singapore.

The destination vectors used in this study are: pAFW (*actin5C* promoter, Flag tagged), pAMW (*actin5C* promoter, Myc tagged), pAVW (*actin5C* promoter, Venus tagged), pAHW (*actin5C* promoter, HA tagged), pTW (pUASt promoter) and pTMW (pUASt promoter, Myc tagged) and pTVW (pUASt promoter, Venus tagged).

**Generation of RNAi resistant UAS-DDB1 construct**

The DDB1 nucleotide sequence targeted by RNAi (VDRC#44974) was altered without changing the protein sequence. A DNA fragment including the modified RNAi-targeted region together flanking ~50-bp sequences was synthesize as a gBLock by IDT (Singapore). The gBlock together with DNA fragments upstream and downstream of the gBlock in *ddb1* CDS were combined by series of PCRs to generate RNAi-resistant full-length CDSs, followed by cloning using the Infusion® HD Cloning Kit (Clontech, Cat#011614) following the manufacturer’s protocol. Primers used to generate an RNAi resistant UAS-DDB1 construct are listed in S3 Table.

**Antibody generation**

To generate anti-DDB1 antibodies, the C-terminal coding sequence (encoding 592–1140 amino acids) of DDB1 tagged with His-Tag at the N-terminus was synthesized by GenScript (Hong Kong) and used as the antigen for antibody generation. Rabbit polyclonal anti-DDB1 antibodies were generated and purified by GenScript (Hong Kong).

Guinea pig polyclonal antisera were raised against a His_6_ fusion protein corresponding to amino acids 841–1116 of the Mahj protein. To produce the His_6_ fusion protein, an 828 bp fragment of *mahj* was ampliﬁed from its cDNA clone SD05932 (*Drosophila* Genomics Resource Center) by PCR using primers 5’-AAGTGCTAGCAAGCTTCCACTCTTTGCC-3’ and 5’-ACACCTCGAGGTTGTAGTTCGAGCTTAG-3’ (restriction sites underlined). The ampliﬁed fragments were cloned into the NheI and XhoI sites of the PET-21a (+) vector. His_6_-Mahj protein was expressed in BL21 *E. coli* cells and puriﬁed on Ni-NTA agarose columns (Qiagen). The purified protein was used to immunize guinea pigs by Cocalico Biologicals Inc. (Reamstown, PA) using standard protocols.

**S2 cell culture, transfection and co-immunoprecipitation**

*Drosophila* S2 cells (CVCL_Z232), a gift from William Chia, were cultured in Express Five serum-free Medium (Thermo Fisher Scientific, Cat #10486-025) supplemented with 2 mM glutamine (Glutamax, Thermo Fisher Scientific, Cat #35050-061). Expression plasmids generated by Gateway cloning were transfected into S2 cells using Effectene Transfection Reagent (QIAGEN, The Netherlands, Cat #301425). S2 cells were collected 48 h after transfection for homogenization. Cells were homogenized in lysis buffer (25 mM Tris pH8, 27.5 mM NaCl, 20 mM, KCl, 25 mM sucrose, 10 mM EDTA, 10 Mm EGTA, 1 mM DTT, 10% vol/vol glycerol, 0.5% Nonidet P40) with Complete Proteases Inhibitors (Complete One, Roche; PNSF 2 mM), phosphatase inhibitors (Sigma, Cat #P5726-1ML), and incubated for 30 min. The supernatants were used for immunoprecipitation with appropriate antibodies overnight at 4°C, followed by incubation with Protein A/G beads for 2 h (Pierces, Rockford, IL). Protein A/G beads were washed with lysis buffer once and with cold PBS three times. Bound proteins were separated by SDS-PAGE and analysed by Western blotting. Mouse anti-Myc (Abcam, Cat #ab32, Clone 9E10), Guinea pig anti-GFP (F. Yu), and mouse anti-Flag (Sigma, Cat #F3165, Clone M2) were used for IP. Primary antibodies used for immunoblotting were: rat anti-HA (1:2000; Roche, Cat #11867423001, Clone 3F10) and mouse anti-Myc (1:2000; Abcam Cat #ab32, Clone 9E10), Rabbit anti-GFP (1:8000; F. Yu) and mouse anti-Flag (1:2000; Sigma, Cat #F3165, Clone M2).

**Protein extraction and immunoblotting**

Approximately 50 larvae of a given genotype were homogenized at 24 h ALH in 150 µl RIPA buffer (1% NP-40; 0.1% deoxycholate, 0.1% SDS, 150 mM NaCl, 50 mM Tris-Cl, pH 8.0) supplemented with protease inhibitors (EDTA-free Complete tablet, Roche, Cat #11873580001) and phosphatase inhibitors (Sigma, Cat #P5726-1ML); phenylmethane sulfonyl fluoride (PMSF) 2 mM (Sigma, Cat #10837091001). Protein lysates were centrifuged for 15 min at 14,000 rpm at 4°C to remove cell debris. The protein extracts were separated by SDS-PAGE and specific proteins were detected by western blot. Three replicas of each experiment were quantitated in ImageJ. Antibodies for western blotting were: guinea pig anti-Mahj (1:5000, this study), and mouse anti-Actin (1:5000, MP Biomedicals, Cat #08691001).

**Quantification of phenotypes in NSCs**

*Drosophila* larval brains were positioned as dorsal side up. Confocal z-stacks were taken, from 40x/1.3 (Zeiss, #420762-9800) and 63x/1.4 (Zeiss, #420782-9900) objective lens, starting from the surface and moving to the deeper layers, for individual larval brain hemispheres, giving around 15–30 slices per z-stack with 3-μm or smaller intervals. ImageJ was used for cell counting from acquired z-stacks. The percentages of Dpn^+^ Mira^+^ NSCs that were positive for EdU, or displayed a cellular process, or that were positive for the mitotic marker PH3 were quantified relative to the total NSCs per brain lobe. The total number of NSCs analysed is given by “t” and the number of brain lobes analysed by “n” for the whole brain analyses and NSC clones in MARCM analysis.

***In vivo* ubiquitination assay in S2 cells**

S2 cells were transfected with pHsp70-hemagglutinin-ubiquitin (HA-Ub; A. Sehgal), Myc-Wts, Flag-Mahj^FL^ or Flag-Mahj^N-term^. The cells were treated with 20 µM MG132 overnight before harvest. At 48 h post-transfection, cells were homogenized with the co-IP lysis buffer, supplemented with protease inhibitors (Complete One, Roche), PMSF 2 mM, MG-132 (20 µM, Sigma, Cat #M7449) and 10 mM Iodoacetamide (IAA, Sigma, Cat #I1149-5G). To induce the expression of HA-ubiquitin plasmid, cells were subjected to heat-shock for 2 h at 37^o^C, followed by 4 h recovery at 25^o^C before harvest. The supernatants were immunoprecipitated with mouse anti-Myc overnight at 4^o^C, followed by incubation with protein A/G beads (Pierce Chemical Co.) for 2 h. Protein A/G beads were washed three times with cold PBS (supplemented with protease inhibitors, 20 uM MG-132 and 10 mM IAA). Bound proteins were separated by SDS-PAGE and analysed by Western blotting with various antibodies.

**Volume analysis of larval brains**

Larval brains of given genotypes were dissected at 96h ALH, fixed and stained with ToPro-3 (DNA) or subjected to immunohistochemistry with other antibodies. LBs were imaged using a Zeiss LSM 710 confocal microscope with 20x/0.8 objective (Zeiss, #420650-9901) to capture the entire CNS in a z-stack with 3 μm intervals. The images were then projected in ImageJ (http://imagej.nih.gov/ij/) to obtain a maximum intensity Z-projection for the entire stack, so that a perimeter that is close to the projected area of a brain hemisphere was drawn manually. The perimeter was used to calculate the sphere volume as an approximate measure for volume of the brain hemisphere.

**Nutrition restriction**

Larvae were transferred to amino acid depleted medium (5% sucrose, 1% Agar in PBS) within 2 h from larval hatching.

**Homology model**

A homology model of *Drosophila* Mahj complexed with *Drosophila* DDB1 (herein referred to as *Dm*Mahj-*Dm*DDB1) was generated by Swiss Model (<https://www.swissmodel.expasy.org/interactive#sequence>) using the Multiple-Target function. The protein sequences of *Drosophila* Mahj and *Drosophila* DDB1 were obtained from Flybase; the proteins share 38% and 49.8% amino acid identity with their human orthologs [36,54]. The crystal structure of the HmDDB1-HmDCAF1 complex (PDB 5JK7[39]) was used as the template for modelling. Chimera Find H-bonds was employed to predict potential H-bonds in the side chains of target residues (R1120, R1123, R1307 and R1343) of *Drosophila* Mahj using Mahj chain C, in which the corresponding residues (R1053, R1056, R1247 and R1248) in PDB 5jk7 are not Ramachandran or side-chain outliers. The potential H-bonds were independently confirmed by the PyMOL Find-polar interactions function. Illustrations of *Dm*Mahj-*Dm*DDB1 and predicted H-bonds were rendered using PyMOL.
